# Analyzing microbial community and volatile compound profiles in the fermentation of cigar tobacco leaves

**DOI:** 10.1007/s00253-024-13043-3

**Published:** 2024-02-29

**Authors:** Mingzhu Zhang, Dongfeng Guo, Haiqing Wang, Guanglong Wu, Yaqi Shi, Jinlong Zhou, Eryong Zhao, Tianfei Zheng, Xingjiang Li

**Affiliations:** 1 Key Laboratory for Agricultural Products Processing, School of Food and Biological Engineering, Hefei University of Technology, Danxia Road 485#, Hefei, 230601 Anhui China; 2 Anhui China Tobacco Anhui Industry Co., Ltd., Huangshan Road 606#, Hefei, 230088 Anhui China

**Keywords:** Cigar tobacco leaves, Microbial community, Volatile compounds, High throughput sequencing, Industrial fermentation

## Abstract

**Abstract:**

Variations in industrial fermentation techniques have a significant impact on the fermentation of cigar tobacco leaves (CTLs), consequently influencing the aromatic attributes of the resulting cigars. The entire fermentation process of CTLs can be categorized into three distinct phases: phase 1 (CTLs prior to moisture regain), phase 2 (CTLs post-moisture regain and pile fermentation), and phase 3 (CTLs after fermentation and drying). These phases were determined based on the dynamic changes in microbial community diversity. During phase 2, there was a rapid increase in moisture and total acid content, which facilitated the proliferation of *Aerococcus*, a bacterial genus capable of utilizing reducing sugars, malic acid, and citric acid present in tobacco leaves. In contrast, fungal microorganisms exhibited a relatively stable response to changes in moisture and total acid, with *Aspergillus*, *Alternaria*, and *Cladosporium* being the dominant fungal groups throughout the fermentation stages. Bacterial genera were found to be more closely associated with variations in volatile compounds during fermentation compared to fungal microorganisms. This association ultimately resulted in higher levels of aroma components in CTLs, thereby improving the overall quality of the cigars. These findings reinforce the significance of industrial fermentation in shaping CTL quality and provide valuable insights for future efforts in the artificial regulation of secondary fermentation in CTLs.

**Key points:**

• *Industrial fermentation processes impact CTLs microbial communities.*

• *Moisture and total acid content influence microbial community succession in fermentation.*

• *Bacterial microorganisms strongly influence CTLs’ aldehyde and ketone flavors over fungi.*

**Supplementary Information:**

The online version contains supplementary material available at 10.1007/s00253-024-13043-3.

## Introduction

Cigars are a specialized type of tobacco product. Traditionally, a cigar is a hand-rolled roll made entirely of cigar tobacco leaves (CTLs) and consists of a wrapper, a binder, and a filler from the outside to the inside. Compared to ordinary cigarettes, cigars are strong, full-bodied, and have a strong aroma. The captivating flavor characteristics of CTLs can be attributed to a combination of the natural environment and the art of fermentation employed in their respective regions of origin (Yin et al. 2019). Cigar production encompasses a critical stage known as fermentation, which integrates both agricultural and industrial processes. The fermentation of tobacco leaves holds immense significance in determining the overall quality of cigars, as it profoundly impacts their physical and chemical properties, as well as the smoking experience (Cai et al. 2019). The objective of agricultural fermentation is to facilitate the degradation and conversion of complex molecules, such as starch and proteins, within tobacco leaves. This process aims to diminish undesirable green and miscellaneous odors, thereby unveiling the leaves’ distinctive aromatic qualities. In contrast, industrial fermentation involves the application of diverse methods and techniques by industrial enterprises. Under specific temperature and humidity conditions, these methods enable the secondary fermentation of cigar tobacco leaves. The ultimate goal is to imbue the leaves with the desired style and characteristics tailored to specific brand requirements. Moreover, the utilization of fermented tobacco leaves in cigar rolling necessitates the involvement of microbial communities in the fermentation process. The metabolic activities of these microbial communities play an irreplaceable role in various aspects, including the degradation of carbohydrates, chlorogenic acid, proteins, Strecker degradation, caramelization reactions, biosynthesis of fatty acids, lipids, amino acids, and the formation of aromatic compounds. These activities contribute to crucial material cycling, energy flow, and aroma development during CTLs fermentation (Banožić et al. 2020). Consequently, the identification of microbial species and the assessment of their diversity throughout different stages of CTLs fermentation, along with the exploration of their functional roles, emerge as paramount factors in enhancing the quality of cigar leaves. This comprehensive understanding aids in reducing the presence of harmful substances and amplifying the expression of unique flavors that characterize exceptional cigars (Chopyk et al. 2017; Smyth et al. 2019).

The advent of sequencing technology has revolutionized the study of microbial communities involved in tobacco fermentation. The application of high-throughput sequencing techniques has yielded valuable insights into the composition of bacterial communities, which are influenced by various factors such as brand, origin, and the fermentation process itself (Malayil et al. 2020; Xing et al. 2021; Liu et al. 2021; Ye et al. 2021; Zhou et al. 2021). In a recent innovative investigation conducted by Chattopadhyay et al., the intricate microbial composition of commercial tobacco products was comprehensively elucidated, unraveling the existence of 89 discrete bacterial genera and 19 fungal genera among diverse tobacco samples. Prominent bacterial species such as *Bacillus*, *Pseudomonas*, and *Staphylococcus* were identified, while *Aspergillus* and *Penicillium* were commonly observed among the fungal species (Chattopadhyay et al. 2021). Furthermore, the metabolic activities of microorganisms in tobacco significantly impact the formation of aromatic compounds, which are pivotal in determining the overall quality of tobacco. Gas chromatography-mass spectrometry (GC–MS) has emerged as the primary method for analyzing volatile metabolites in tobacco in recent studies (Vu et al. 2021; Qin et al. 2021). Prominent metabolites frequently observed encompass tobacco alkaloids, aldehydes, ketones, alcohols, and alkanes (Zelinkova and Wenzl 2021).

However, our current knowledge of cigar fermentation remains incomplete, with a lack of comprehensive analysis that combines metabolomics and microbial diversity. While recent studies employing multi-omics approaches have provided valuable insights into tobacco products (Tsaballa et al. 2020; Tyx et al. 2020), the integration of metabolomic profiling and the examination of bacterial-fungal microbial diversity during CTLs fermentation are still limited. Therefore, the objective of this study was to provide a comprehensive understanding of the distinct stages of industrial fermentation in CTLs through the collection of samples from the Yunnan region. The analysis encompassed a comparative assessment of community structure characteristics and a systematic evaluation of the composition of metabolic products. Ultimately, the study established correlations between the predominant microorganisms found in cigars and the key metabolic products during the fermentation process. These research findings offer valuable theoretical support for understanding the characteristics of microbial communities and metabolic patterns in the industrial fermentation environment of CTLs.

## Materials and methods

### Sample preparation

CTLs samples utilized in this study were generously provided by the Technical Center of Anhui China Tobacco Industry Co., Ltd., ensuring a reliable and representative sample set. To delineate the temporal progression of fermentation, samples were procured at six distinct time intervals: UFCL (unfermented cigar leaves) before moisture reacquisition, MRCL (moisture-regained cigar leaves) post moisture reacquisition, FCL (fermented cigar leaves) subsequent to moisture reacquisition and fermentation, FTCL (first turn cigar leaves) after the initial turn, STCL (second turn cigar leaves) post second turn, and DCL (dried cigar leaves) after the completion of fermentation and drying, as elaborated in Table S1. From each time point, samples were collected from distinct positions within the fermentation pile, namely the upper, middle, and lower layers, as depicted in Figure S1. To ensure statistical robustness and reduce spatial variability, a total of 15 samples from the three layers were combined to create a single biological replicate. These replicates were carefully transferred to sterile plastic bags to maintain sample integrity. Subsequently, all samples were rapidly frozen in liquid nitrogen and ground to a fine powder. To maintain the integrity of the samples, stringent measures were taken to ensure their preservation. The samples were meticulously stored at temperatures of − 20 °C and − 80 °C, catering to their specific analytical requirements for physicochemical analysis and DNA extraction, respectively. These rigorous sample collection and handling protocols were implemented with the purpose of securing precise and dependable outcomes, thereby facilitating a comprehensive exploration into the physicochemical properties and microbial diversity of CTLs throughout the fermentation process.

### Determination of physicochemical factors

In our comprehensive exploration of fermentation processes in diverse environments, we meticulously quantified and evaluated five crucial fermentation parameters: moisture, chroma, total sugar content, reducing sugar content, and titratable acidity. The moisture of the CTLs was determined using a gravimetric method, by drying samples to a constant weight at 105 °C for at least 3 h. Titratable acidity, a vital indicator of fermentation progress, was quantified through a meticulous NaOH titration method employing phenolphthalein as a sensitive indicator (Liu et al. 2017; Tan et al. 2019; Zhang et al. 2020b). To assess chroma, a fundamental characteristic of CTLs, we employed a precise colorimeter measurement. Furthermore, the determination of total and reducing sugar contents was conducted using the established and reliable Fehling’s reagent method. These meticulous and established techniques ensured the acquisition of accurate and reliable data, thus providing valuable insights into the fermentation dynamics of CTLs (Zhang et al. 2021b).

### Profiling volatile compounds in cigar tobacco leaves

The CTLs samples (0.5 g) were immersed in an 8 mL saturated NaCl solution. To ensure accurate quantification, we added 1 µL of the internal standard (phenylmethyl acetate) at a concentration of 128.75 µg/µL to a 20 mL vial, which was securely sealed with a silicone septum. In a temperature-controlled water bath with magnetic agitation, the vials were subjected to regulated extraction conditions. For the first 20 min, the samples were kept at a temperature of 70 °C. Following that, a static water bath extraction was performed for 35 min at the same temperature of 70 °C. Using DVB/CAR/PDMS-coated fibers (50/30-m; Supelco Inc., Bellefonte, PA, USA), the headspace solid-phase microextraction method was utilized to extract flavor components from CTLs samples. To conduct the gas chromatography-mass spectrometry (GC–MS) analysis, we employed the Shimadzu GCMS-QP2010 apparatus. After the extraction, the fibers were injected into the gas chromatograph’s injection port (250 °C) for 5 min to allow the analytes to desorb. After that, the analytes were separated and analyzed using a DB-5MS column (60 m × 1 mm i.d., 0.32-µm film thickness; J&W Scientific, CA, USA). The GC–MS settings were as follows: helium as the carrier gas at a constant flow rate of 0.8 mL/min, a temperature ramp to 180 °C at a rate of 3 °C/min, and a 2-min hold at 180 °C. Subsequently, the temperature was raised to 260 °C at a rate of 6 °C/min and held there for 2 min. The mass spectrometer was set to electron ionization at 70 eV and an ion source temperature of 230 °C. Full-scan acquisition was carried out in the mass range of 35 to 450 amu to allow for in-depth examination of the compounds. The mass spectrometric information, retention indices (determined using n-alkanes), and mass spectra of unknown compounds were compared with the National Institute of Standards and Technology (NIST 14, https://www.nist.gov) mass spectra library (match threshold 80). For semi-quantification, volatiles’ relative peak areas in relation to the reference substance phenylmethyl acetate were calculated (Zheng et al. 2022a).

### DNA extraction, PCR, and bioinformatics analysis

To ensure comprehensive analysis of bacterial and fungal diversity in CTLs samples, we conducted three independent biological replicates. The purity and concentration of DNA were assessed using the NanoDrop2000 system, while DNA integrity was evaluated through agarose gel electrophoresis. To facilitate high-throughput sequencing, the cigar samples were pulverized using a homogenizer and filtered through a 40-mesh sieve to retain the desired material. Subsequently, microbial community genomic DNA was extracted from 18 samples using the HiPure Soil DNA Kit (Magen, Guangzhou, China) following the manufacturer’s instructions. The extracted DNA was visualized on a 1% agarose gel, and the DNA concentration and purity were determined using a NanoDrop 2000 UV–Visible spectrophotometer (Thermo Scientific, Wilmington, USA).

After conducting a comprehensive review of relevant literature (Zhang et al. 2020a, 2020c; Dan et al. 2020), we discerned that targeting the V4 region for amplification benefits data modeling and analytical processes, thereby offering a robust framework for analyzing and annotating microbial diversity. Specifically, we assessed bacterial diversity by amplifying the V4 hypervariable region of the 16S rRNA gene using the primer set 515F (5′-GTGYCAGCMGCCGCGGTAA-3′) and 806R (5′- GGACTACNVGGGTWTCTAAT-3′). For fungal diversity analysis, the internal transcribed spacer (ITS) region was amplified using the primer set ITS1F (5′-CTTGGTCATTTAGAGGAAGTAA-3′) and ITS2 (5′-GCTGCGTTCTTCATCGATGC-3′). The total genomic DNA underwent high-throughput sequencing using the cutting-edge Miseq desktop sequencer (2 × 300 bp; Illumina MiSeq PE300, San Diego, CA, USA), courtesy of Guangzhou GenDenovo Honour Biotechnology Co., Ltd. All generated raw sequences in this study were processed utilizing the USEARCH algorithm. This algorithm is instrumental in clustering sequences into operational taxonomic units (OTUs), a key step in analyzing biological sequence data. The primary advantage of USEARCH lies in its capacity for rapid and accurate clustering of similar sequences (Kopylova et al. 2016). To unravel the intricate taxonomic composition, the representative sequences of bacterial OTUs were meticulously aligned against the comprehensive Silva 132_16S rRNA database (https://www.arb-silva.de/). Similarly, a thorough taxonomic assignment was made possible by comparing the representative fungal OTUs sequences to the extensive UNITE fungal ITS database (https://unite.ut.ee/).

### Data analysis

Multivariate analysis was employed to examine significant differences among microorganisms and volatile compounds, utilizing SIMCA software (version 14.0). This analysis encompassed techniques such as partial least squares discriminant analysis (PLS-DA), principal component analysis (PCA), and variable importance in projection (VIP). PLS-DA is a supervised multivariate statistical analysis technique that combines metabolite changes with experimental grouping information through regression models while reducing dimensions. To assess the model’s quality, we utilize metrics such as R2X, R2Y, and Q2. R2X and R2Y indicate the percentage of variance in X and Y matrices explained by the PLS-DA model, respectively. Q2, calculated through cross-validation, evaluates the predictive ability of the PLS-DA model. Typically, R2 and Q2 values above 0.5 suggest a well-fitted model; however, values slightly below 0.5 can be acceptable, indicating a weaker model. However, supervised classification models like PLS-DA can be prone to overfitting. To address this, we conduct permutation tests to assess overfitting. If the regression line of Q2 intersects the *y*-axis at a point less than zero, it indicates the absence of overfitting, signifying the reliability of the PLS-DA model. In addition, correlation analysis was carried out using the SPSS software (version 25.0). To obtain insight into the general community structure, Omicsmart (http://www.omicsmart.com) was used to perform principal coordinate analysis (PCoA) and variation partition analysis (VPA). The heatmap was generated and correlation analysis was performed using ChiPlot (https://chiplot.online/). Gephi software (version 0.10.1) was used for visual network analysis.

## Results

### Analysis of physicochemical parameters for different cigar tobacco leaves

**Fig. 1:**
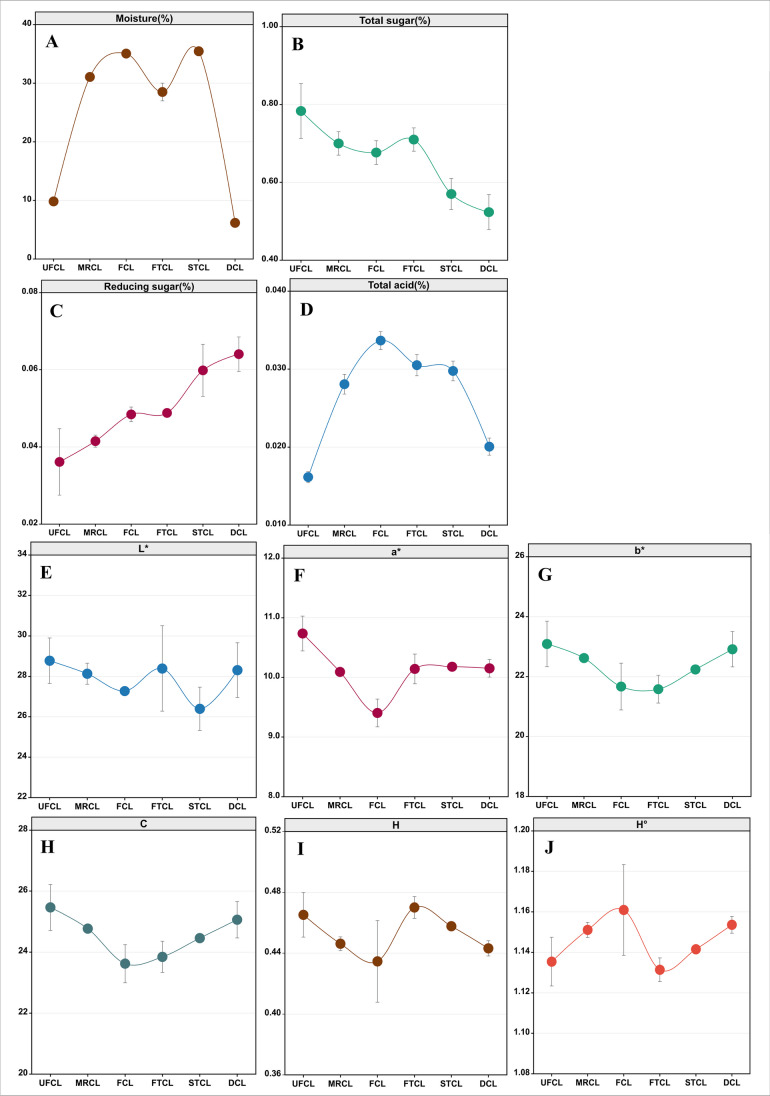
Changes in the physicochemical indices during different stages of fermentation for CTLs. **A** Moisture; **B** total sugar; **C** reducing sugar; **D** total acid; and chromatic parameters **E** L*, **F** a*, **G** b*, **H** *C*, **I** *H*, and **J** *H*°. The stages of fermentation are denoted as UFCL (unfermented cigar leaves) representing leaves before moisture reacquisition, MRCL (moisture-regained cigar leaves) indicating leaves post moisture reacquisition, FCL (fermented cigar leaves) denoting leaves subsequent to moisture reacquisition and fermentation, FTCL (first turn cigar leaves) representing leaves after the initial turn, STCL (second turn cigar leaves) indicating leaves post second turn, and DCL (dried cigar leaves) representing leaves after the completion of fermentation and drying

During the CTL fermentation process, we investigated the dynamic changes in moisture content, chroma, total sugar, reducing sugar concentration, and total acidity (Fig. 1). After rehydration, the moisture content of CTLs increased fast, with the moisture content of UFCL to MRCL increasing from 9.82 to 31.06%. As the fermentation time progressed, a decreasing trend in total sugar content and a gradual increase in reducing sugar content were observed. The reduction in chemical component concentration in tobacco leaves is attributed to enzyme activity and microbial metabolism. Specifically, during the fermentation process, activities such as amylase increase, leading to starch hydrolysis and the formation of reducing sugars, as demonstrated in previous studies (Yamaguchi et al. 2013). The total sugar concentration, on the other hand, does not grow, possibly because bacteria use these sugars as resources to support their metabolism. In contrast, the total acidity of CTLs was initially low in the UFCL samples at the beginning of fermentation and then increased before drying. The total color difference (▲E*) represents the overall change in color during agricultural product processing or storage, incorporating the three fundamental color parameters: L*, a*, and b*. A higher value indicates a more pronounced color change. The trends of the tobacco leaf color parameters L*, a*, b*, *C*, *H*, and *H*° during the fermentation process are depicted in Fig. 1. It can be observed that there are significant differences in the tobacco leaf color parameters during fermentation. With the progression of fermentation, the L*, a*, b*, and *C* values initially decrease and then increase, with the most substantial changes occurring after the first turning of the fermentation pile. Subsequently, the magnitude of the changes gradually diminishes, reaching a stable state. As the fermentation progresses, the *H* value follows a pattern of initial decrease, subsequent increase, and final decrease. The highest value is observed during the first turning of FTCL, followed by a gradual decline. Conversely, the *H*° value demonstrates an initial increase, followed by a decrease, and then another increase. The lowest value is observed during the first turning of FTCL, after which there is a gradual rise.

We performed regression analysis to examine the relationship between the physicochemical parameters of CTLs during industrial fermentation and the total color difference (▲E*). Using moisture content, total sugar, reducing sugar, and total acidity as dependent variables and ▲E* as the independent variable, we established regression equations for each physicochemical indicator at different fermentation stages (Figure S3). The ▲E* value exhibited a gradual upward trend (Figure S2), indicating a progressive darkening of the color in CTLs during fermentation. The regression analysis revealed that moisture content, reducing sugar, and total acidity had a direct and positive impact on the ▲E* value, while total sugar content had a direct and negative impact. In summary, the physicochemical parameters directly influenced the color changes of CTLs throughout the fermentation process.

### Analysis of volatile compounds

The profiles of volatile compounds (VOCs) in CTLs were detected using GC–MS to explore the impact of CTLs fermentation on volatile flavors. Eight aldehydes, 25 ketones, 3 esters, 7 alkanes, 4 terpenes, 2 nicotinoids, 5 alcohols, 2 phenols, and 7 additional volatile scents were found during fermentation. In all CTLs samples, the bulk of volatile chemicals (39.68% of all volatile components) were ketones (Figure S4).

**Fig. 2:**
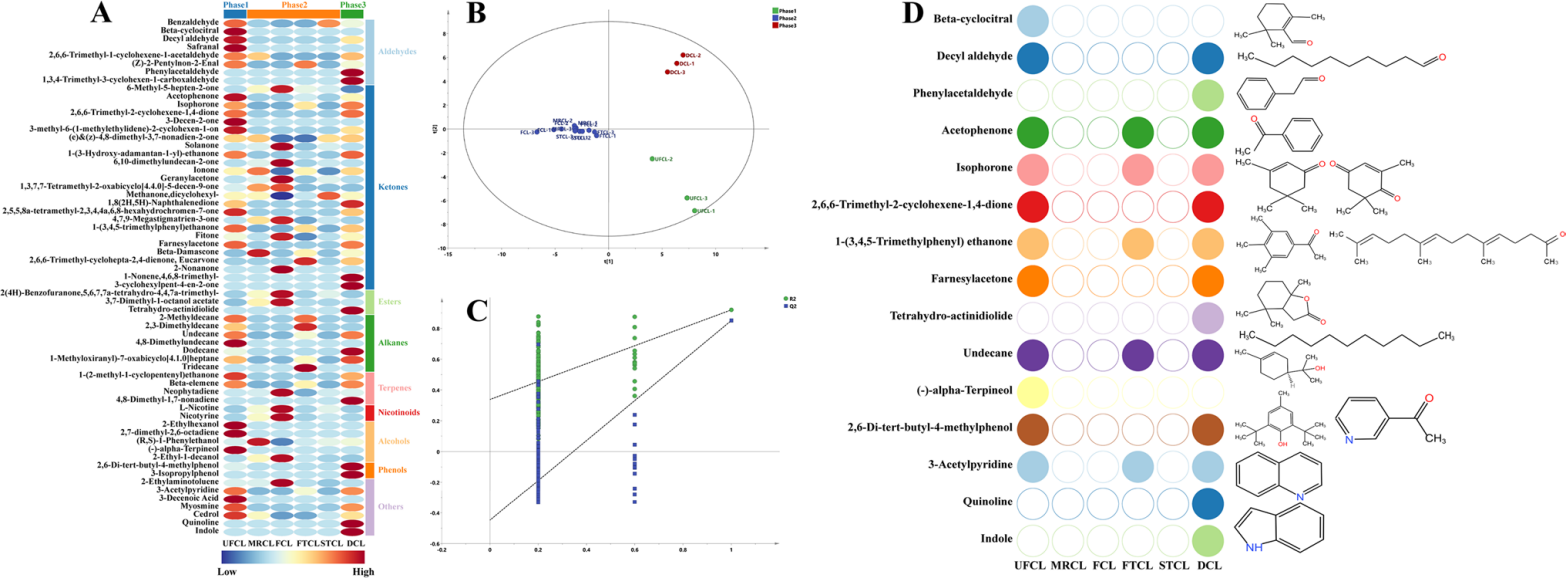
**A** Heatmap analysis was performed to examine the volatile compounds in CTLs during fermentation; **B** PLS-DA score plots of volatile flavors were generated to analyze their characteristics; **C** permutation tests were performed to compare the three different stages of fermentation, and **D** key volatile compounds were identified using PLS-DA, with a VIP score of > 1.0 and an OAV score of ≥ 1.0, indicating their significance and contribution. CTLs are classified into three phases: phase 1 (before moisture regaining, including UFCL), phase 2 (after moisture regaining, including MRCL, FCL, FTCL, and STCL), and phase 3 (after fermentation and drying, including DCL)

The heatmap analysis results show that the metabolic profiles of fermented CTLs have various stages, as shown in Fig. 2A. In phases 1 and 3, there was a notable increase in both the diversity and concentration of metabolites compared to phase 2. Among these, ketone compounds were particularly variable. Out of the 25 ketone compounds identified, 16 flavor components showed significant fluctuations throughout these phases. The three VOCs with the most marked variability were 4,7,9-megastigmatrien-3-one, geranylacetone, and solanone. Their relative concentrations varied significantly across the three phases, with ranges of 77.50 to 126.35 µg/µL for 4,7,9-megastigmatrien-3-one, 40.83 to 77.81 µg/µL for geranylacetone, and 49.16 to 85.83 µg/µL for solanone. Besides, volatile nicotine and neoplantene emerge as the predominant compounds in CTLs, constituting 77.81 to 94.76% of the total volatiles. Throughout the fermentation process, the relative content of volatile nicotine initially rises, then declines (50.87% vs 80.12% vs 51.98%), whereas neoplantene exhibits a decrease followed by an increase (26.94% vs 11.35% vs 28.56%). This fluctuation in volatile nicotine may be attributed to the conversion of alkaloids in CTLs and their subsequent release into the air during deep fermentation. Neoplantene, a degradation product of chlorophyll, itself lacks a strong aroma or fragrance but significantly influences sensory attributes such as aroma quality, smoothness, and transmittance.

Based on the analysis of 63 VOCs as the dependent variable and different fermentation stages as the independent variable, the results of PLS-DA (Fig. 2B) and PCA (Figure S4) demonstrate effective discrimination of CTLs at various fermentation stages. PLS-DA is a supervised multivariate statistical analysis technique that combines metabolite changes with experimental grouping information through regression models while reducing dimensions. To assess model’s quality, we utilize metrics such as R2X, R2Y, and Q2. The goodness of fit indices for the independent variables, represented by R2x (0.724), and the dependent variables, represented by R2y (0.961), indicate satisfactory model fitting. Additionally, model’s prediction index, Q2 (0.926), surpasses the threshold of 0.5, further supporting the reliability of the model (Yun et al. 2021). The validity of the model is confirmed by performing 200 permutation tests, as depicted in Fig. 2C, where the intersection between the Q2 regression line and the *y*-axis is negative, indicating the absence of overfitting. As a result, these data can be used to identify and analyze different fermentation phases in CTLs. Furthermore, a VIP score threshold of > 1.0 (Figure S4) was employed in combination with the screening criterion of olfactory activity value (OAV) > 1.0 (Table S3) to assess the impact strength and explanatory power of differential metabolite accumulation differences on the classification and discrimination of samples in each group. In total, 15 metabolites exhibited substantial differences among the three groups and made significant contributions to the overall aroma of CTLs. These metabolites are beta-cyclocitral, decyl aldehyde, phenylacetaldehyde, acetophenone, isophorone, 2,6,6-trimethyl-2-cyclohexene-1,4-dione, 1-(3,4,5-trimethylphenyl) ethanone, farnesylacetone, tetrahydro-actinidiolide, undecane, (-)-alpha-terpineol, 2,6-di-tert-butyl-4-methylphenol, 3-acetylpyridine, quinoline, and indole. Figure 2D depicts the temporal fluctuations of these 15 metabolites during the fermentation process. In Fig. 2D, distinctive circular shapes of various colors represent 15 different types of differential metabolites, while the unfilled circles denote volatile metabolites that were not detected in this specific sample. It is readily apparent that 11 and 13 differential metabolites were identified in UFCL and DCL, respectively, providing further evidence of the higher diversity in both types and quantities of metabolites during phase 1 and phase 3 in comparison to phase 2. Notably, among the detected differential metabolites, ketones constitute 45.45% and 38.46% in UFCL and DCL, respectively, showcasing a more prominent variability.

### Microbial community composition diversity and differences

The researchers observed the industrial fermentation process of CTLs to evaluate the microbial community makeup among different groups. Following the application of quality control procedures, 18 samples yielded 3,042,137 high-quality sequences that represented communities of bacteria. The number of sequences in the sample ranged from 74,366 to 219,738. Also obtained were 2,300,041 high-quality reads from fungi, with read counts per sample ranging from 111,954 to 136,222. The sequence numbers retrieved were sufficient to reach saturation, according to the goods coverage analysis (Table S2).

**Fig. 3:**
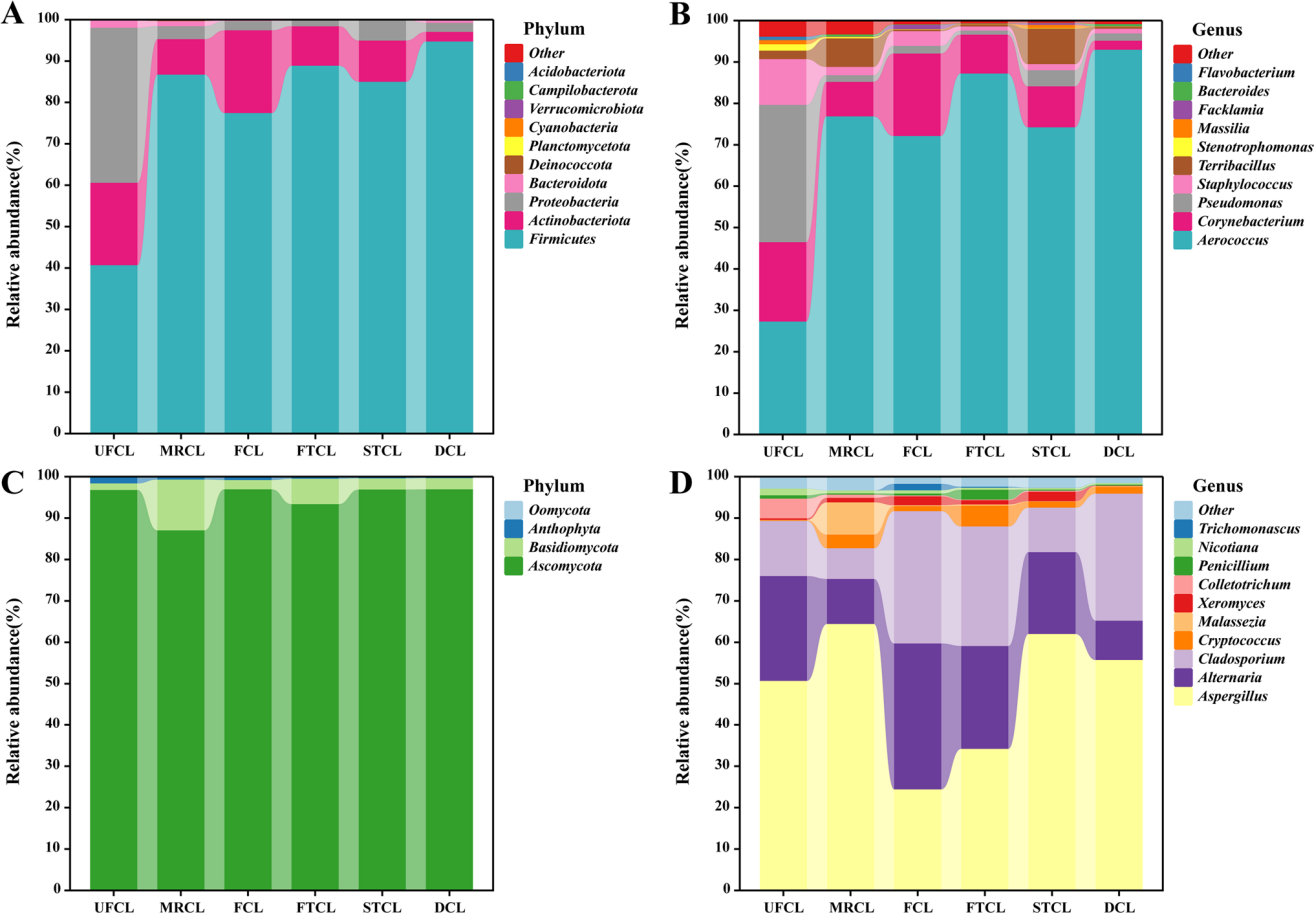
The distribution of the microbial community in CTLs at various stages of fermentation. Bacteria (**A**) and fungus (**C**) at the phylum level; bacteria (**B**) and fungi (**D**) at the genus level

At the phylum level, the bacterial community was dominated by members of the Firmicutes phylum, which accounted for 94.73% of the relative abundance at the final stage of fermentation (Fig. 3A). Moving down to the genus level, during the initial stages of fermentation (UFCL/Phase1), *Corynebacterium*, *Pseudomonas*, *Staphylococcus*, and *Stenotrophomonas* displayed higher abundance. However, following the rehydration of CTLs, *Aerococcus* emerged as the dominant genus and maintained its prevalence throughout the fermentation process (Fig. 3B). Shifting the focus to the fungal aspect, the Ascomycota phylum dominated the fungal population at the start of fermentation, accounting for 96.82% of the total microbial abundance (Fig. 3C). At the genus level (Fig. 3D), prior to and after moisture regaining (UFCL/MRCL), *Aspergillus* exhibited dominance, with relative abundances of 50.68% and 64.44% in UFCL and MRCL, respectively. *Alternaria* and *Cladosporium* were the dominant genera in the accumulated CTLs pile, accounting for relative abundances of 35.28% and 31.96%, respectively. Notably, a slight increase in fungal diversity was observed in the FTCL during fermentation, with *Aspergillus*, *Alternaria*, and *Cladosporium* emerging as the dominant genera, comprising abundances of 34.22%, 24.90%, and 28.86%, respectively. *Aspergillus* maintained dominance in both STCL and DCL, with abundances of 62.01% and 55.73%, respectively.

**Fig. 4:**
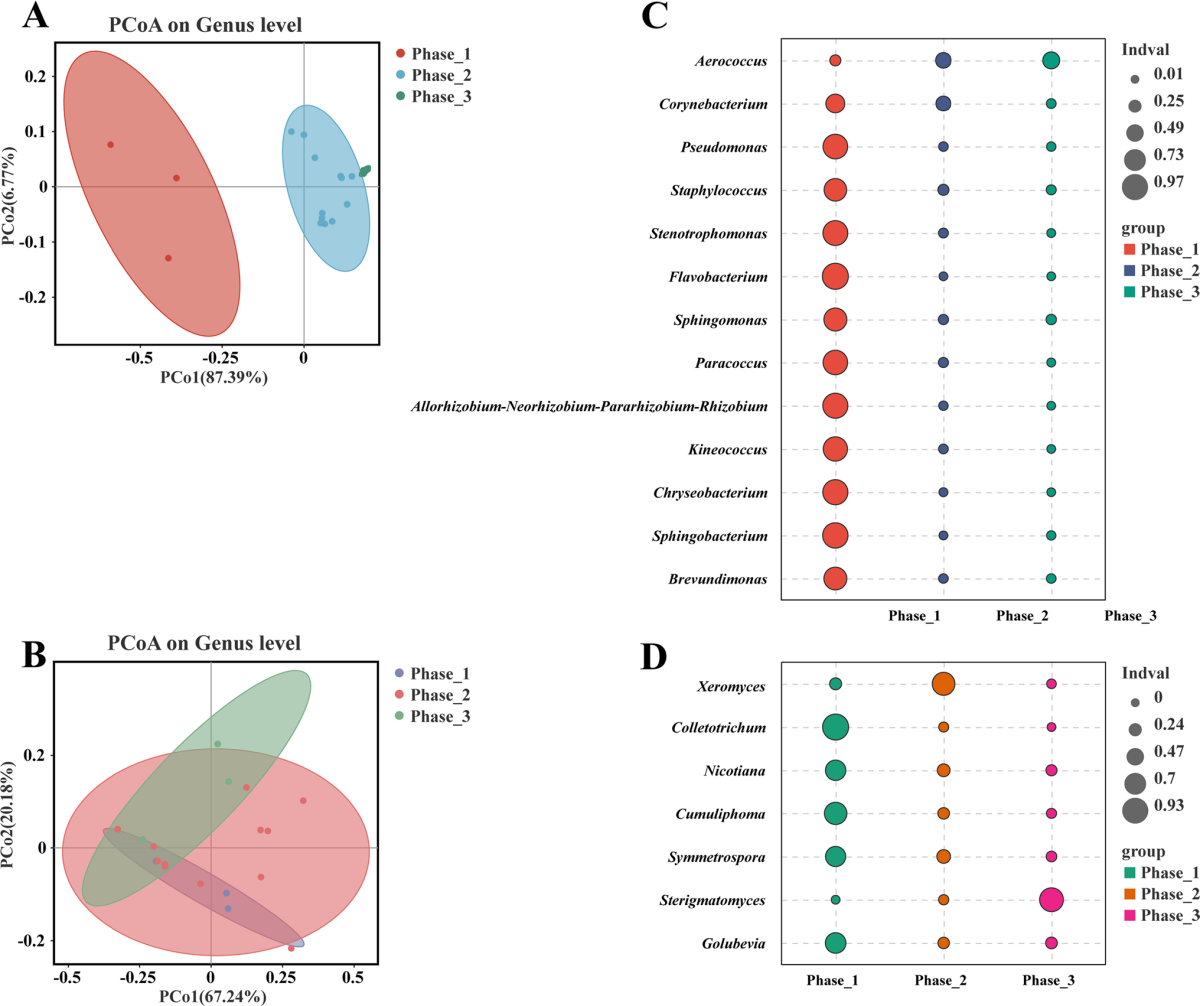
The dynamics of the microbial community in CTLs at various stages of fermentation. The following are the results of principal coordinate analysis (PCoA) of bacterial (**A**) and fungal (**B**) communities using the Bray–Curtis distance matrix; indicator analysis; and cross-validation statistical tests: bacteria (**C**) and fungi (**D**)

By analyzing *α*-diversity and *β*-diversity, microbial richness and diversity can be calculated. The fermentation process can be separated into stages 1, 2, and 3 based on the results of the diversity and cluster analysis. Shannon and Chao index values of CTLs showed a relatively high level of diversity in phase 1 (pile fermentation: CTLs before moisture recovering). In phase 2 (pile fermentation: CTLs after moisture regaining, fermentation, and turning), Shannon’s diversity index and Chao index values initially decreased and subsequently either increased or remained relatively stable. In phase 3 (CTLs after fermentation and drying), Shannon’s diversity index and Chao index values of CTLs all decreased to a comparatively lower level. Moreover, to explore the divergent microbial communities, we conducted *β*-diversity analysis for the three groups. As depicted in Fig. 4A–B, the principal coordinates analysis based on the Bray–Curtis distance matrix demonstrated the distinct separation of bacterial and fungal communities into three distinct clusters. The different fermentation processes had specific impacts on both bacterial and fungal populations. However, when compared to the fungal PCoA distribution trend (Fig. 4B), the differentiation of bacterial microbiota at different stages was more pronounced (Fig. 4A). These disparities in bacterial microbial community composition might be more substantial, indicating that bacteria could be pivotal in the dynamic changes observed in the metabolite profile during fermentation stages.

Indicator value analysis was conducted to compare the species richness across the three stages of CTLs and identify the significant differences in the studied genera. A comprehensive assessment of bacterial genera revealed a total of 13 genera that exhibited noteworthy significance as indicators for distinct fermentation stages. Similarly, for fungal genera, a total of 7 genera demonstrated significant indicator values (indicator value *p* < 0.01). Remarkably, the microbial genus assemblages indicative of phase 1, 2, and 3 fermentation stages, as classified based on CTLs’ diverse industrial fermentation processes, remained consistently present throughout the sampling period. However, as quantified by the coefficient of variation, certain time points displayed higher relative abundances and lower variation (Fig. 4C–D).

### Relationship between the microbial community, physicochemical characteristics, and volatile compounds

**Fig. 5:**
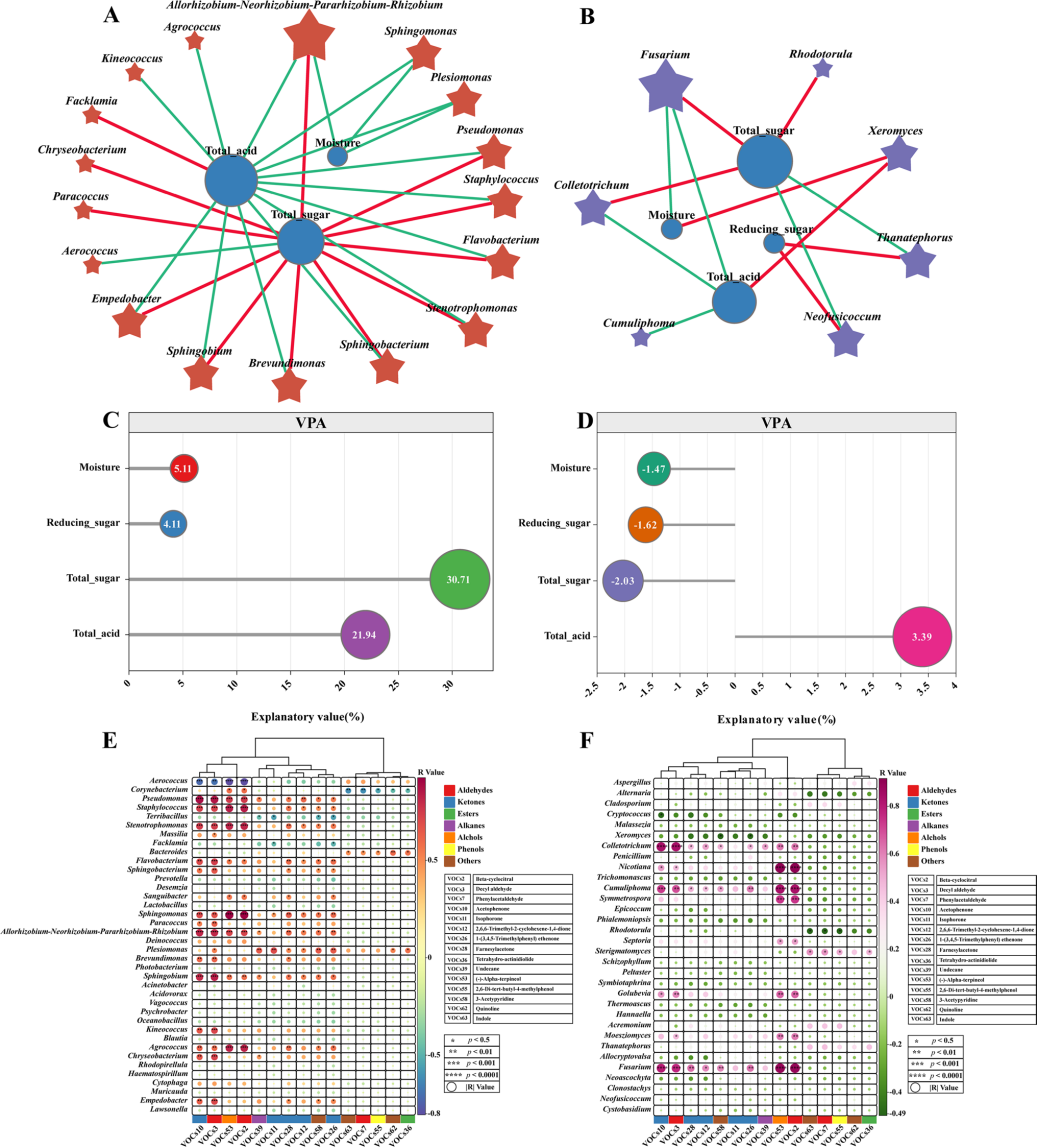
The correlation network between selected bacteria and physicochemical indicators (**A**), and the correlation network between selected fungi and physicochemical indicators (**B**). Pearson’s correlation coefficient (*R* > 0.5, *p* < 0.05) was used to calculate the selected bacteria or fungi; variation partitioning analysis of the relative contributions of moisture, reducing sugar, total sugar, and total acid to variation in CTLs bacterial β-diversity (**C**); and variation partitioning analysis of the relative contributions of moisture, reducing sugar, total sugar, and total acid to variation in CTLs fungal β-diversity (**D**). Heat map of correlation between bacterial (**E**) with fungal (**F**) and VOCs; blue and green represent positive correlation, red and purple represent negative correlation, and the size of the circles represents the magnitude of the |*R*| value; significant correlations were expressed by *, **, ***, and ****, which were *p* < 0.05, *p* < 0.01, *p* < 0.001, and *p* < 0.0001, respectively. VOCs2, beta-cyclocitral; VOCs3, decyl aldehyde; VOCs7, phenylacetaldehyde; VOCs10, acetophenone; VOCs11, isophorone; VOCs12, 2,6,6-trimethyl-2-cyclohexene-1,4-dione; VOCs26, 1-(3,4,5-trimethylphenyl) ethenone; VOCs28, farnesylacetone; VOCs36, tetrahydro-actinidiolide; VOCs39, undecane; VOCs53, (-)-alpha-terpineol; VOCs55, 2,6-di-tert-butyl-4-methylphenol; VOCs58, 3-acetylpyridine; VOCs62, quinoline and VOCs63, indole

In all CTLs samples, Pearson correlation analysis was carried out (*R* > 0.5, *p* < 0.05; Fig. 5A–B) to investigate any potential relationships between the dominating genera and four important environmental parameters (moisture, total acid, total sugar, and reducing sugar content). The results unveiled intriguing associations between these factors and the microbial composition. Notably, a positive correlation was observed between total sugar and bacterial genera. Conversely, moisture and total acid displayed negative correlations with all bacterial genera, except for *Facklamia* (Fig. 5A). It is important to note that fungi were affected by environmental conditions differently than bacteria were. Both total sugar and reducing sugar exhibited positive correlations with five selected fungal genera, while moisture and total acid displayed negative correlations with all genera, with the exception of *Xeromyces* (Fig. 5B). These correlation analyses may offer a new insight into the complex dynamics of CTLs fermentation by highlighting the complicated interplay between physicochemical characteristics and microbial communities.

The impact of moisture, reducing sugar, total sugar, and total acid on microbial community variation is illustrated in a bar diagram (Fig. 5C–D). The comprehensive analysis of all variables accounted for 61.87% and 8.51% of the variation in the bacterial and fungal communities of CTLs, respectively. Among the bacterial community, the major contributor was total sugar, which accounted for 30.71% of the variation. Additionally, total acid (21.94%) made a substantial contribution, while moisture (5.11%) and reducing sugar (4.11%) had comparatively smaller effects on bacterial β-diversity in CTLs. Regarding the fungal community, total acid (3.39%) emerged as the primary contributing factor. On the other hand, moisture (− 1.47%), reducing sugar (− 1.62%), and total sugar (− 2.03%) displayed negative contributions to the fungal community variation.

To explore the intricate relationship between bacteria and flavor development during fermentation, we conducted a comprehensive analysis using a correlation heat map and cluster analysis. The correlations between bacterial and fungal communities and the metabolite profiles of fermented CTLs may be divided into three different groups, according to our findings, which are shown in the hierarchical cluster analysis (HCA) results (Fig. 5E–F). In terms of bacterial microorganisms, cluster 1 consisted of VOCs10 (acetophenone), VOCs3 (decyl aldehyde), VOCs53 ((-)-alpha-terpineol), and VOCs2 (beta-cyclocitral). These compounds exhibited significant positive correlations with *Pseudomonas*, *Staphylococcus*, *Stenotrophomonas*, *Flavobacterium*, *Sphingomonas*, *Allorhizobium-Neorhizobium-Parararhizobium-Rhizobium*, *Sphingobium*, and *Agrococcus* were also significantly positively correlated while showing significant negative correlations with *Aerococcus*. Cluster 2 consisted of VOCs39 (undecane), VOCs11 (isophorone), VOCs28 (farnesylacetone), VOCs12 (2,6,6-trimethyl-2-cyclohexene-1,4-dione), VOCs58 (3-acetylpyridine), and VOCs26 (1-(3,4,5-trimethylphenyl) ethenone). These compounds displayed significant positive correlations with the nine bacterial genera mentioned earlier, although with smaller *R*-values and weaker correlations compared to cluster 1. Cluster 3, consisting of VOCs63 (indole), VOCs7 (phenylacetaldehyde), VOCs55 (2,6-di-tert-butyl-4-methylphenol), VOCs62 (quinoline), and VOCs36 (tetrahydro-actinidiolide), exhibited a distinct pattern. Notably, cluster 3 showed a significant negative correlation with *Corynebacterium* and a significant positive correlation with *Bacteroides*, distinguishing it from the other clusters. Regarding fungal microorganisms, cluster 1 encompassed VOCs10, VOCs3, VOCs28, and VOCs12, which demonstrated significant positive correlations with *Colletotrichum*, *Cumuliphoma*, and *Fusarium*. Cluster 2 comprised VOCs58, VOCs11, VOCs26, VOCs39, VOCs53, and VOCs2. These compounds exhibited some positive correlations with the aforementioned three fungal genera, with VOCs53 and VOCs2 showing significant positive correlations with *Nicotiana*, *Symmetrospora*, *Septoria*, *Golubevia*, and *Moesziomyces*. Cluster 3, consisting of VOCs63, VOCs7, VOCs55, VOCs62, and VOCs36, displayed distinct associations. Notably, the clustering pattern in cluster 3 for bacterial genera was consistent, suggesting a consistent trend in the effects of these VOCs on the microflora. Cluster 3 exhibited a significant positive correlation with *Sterigmatomyces* and a significant negative correlation with *Rhodotorula*.

### Metabolic map of important volatile compounds during the industrial fermentation of cigar tobacco leaves

**Fig. 6:**
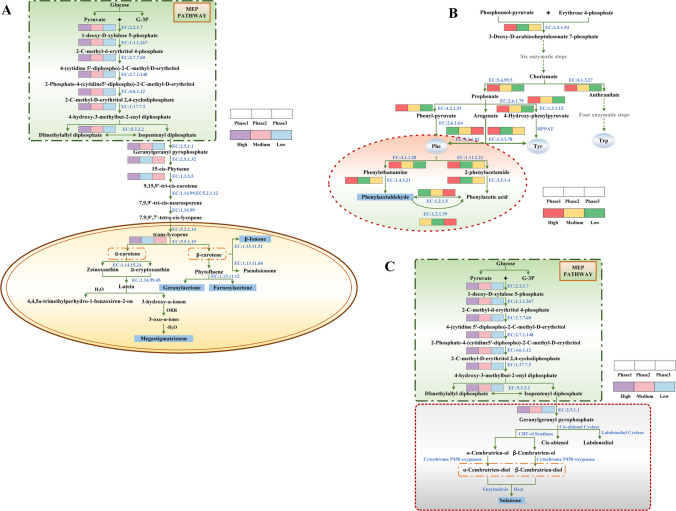
Metabolic map of important VOCs during the industrial fermentation of CTLs, **A** the pathway of carotenoid biosynthesis and degradation, **B** aromatic amino acid biosynthesis leading to Trp, Tyr, and Phe in plants, and **C** biosynthetic and degradation pathway of cembranoid diterpenes. The MEP pathway is represented by the pathway in the rectangle above, which produces substrates for carotenoid synthesis. The pathway in the bottom ellipse illustrates carotenoids’ synthesis and degradation in plastids. Enzymes are depicted in blue

To gain deeper insights into the association between microorganisms and VOCs, we employed the Kyoto Encyclopedia of Genes and Genomes (KEGG) database and relevant literature to predict the metabolic network of significant VOCs during the industrial fermentation of CTLs. Analysis of the metabolic pathways using the KEGG and MetaCyc databases revealed that the synthesis and biosynthesis pathways of key flavor compounds were generally consistent. Figure 6 illustrates the impact of the bacterial community on the profiles of VOCs through the modulation of relevant enzyme activities.

The VOCs present in CTLs can be categorized into distinct groups based on their origin, including benzene carotenoid degradation products, phenylalanine degradation products, cypress-like substances, and chlorophyll degradation products (neophytadiene). As depicted in Fig. 6A, carotenoids are synthesized through the condensation of isopentenyl diphosphate (IPP) and dimethylallyl diphosphate (DMAPP), which are generated via the 2-C-methyl-d-erythritol 4-phosphate pathway (MEP) (Klee and Tieman 2013). Crucial enzymes involved in this pathway, such as EC: 2.2.1.7 (1-deoxy-d-xylulose-5-phosphate synthase) and EC: 1.1.1.267 (1-deoxy-d-xylulose 5-phosphate reductoisomerase), play a fundamental role in determining the production rate of IPP. It is important to note that the content and composition of carotenoids in plants are not solely influenced by the biosynthesis pathway but also by the degradation pathway. Carotenoids serve as essential precursors for various bioactive compounds. They can undergo enzymatic cleavage, primarily catalyzed by EC: 1.13.11.68 (carotenoid cleavage dioxygenases) and EC: 1.13.11.51 (9-cis-epoxycarotenoid dioxygenases), or non-enzymatic reactions initiated by reactive oxygen species (ROS). Carotenoid degradation products, such as *β*-ionone, geranylacetone, farnesylacetone, and megastigmatrienone, are significant VOCs found in CTLs. Enhancing the levels of VOCs through increased carotenoid degradation may be considered an effective approach due to the positive association between carotenoid degradation and flavor formation.

Phenylalanine (Phe) and tryptophan (Trp) are considered essential aromatic amino acids, whereas tyrosine (Tyr) is deemed non-essential (Fürst and Stehle 2004). The synthesis of these three amino acids begins with the shikimate pathway, which converts phosphoenolpyruvate and erythrose 4-phosphate into chorismate, which is then converted into Phe and Trp via the aromatic amino acid biosynthetic pathways (Tzin and Galili 2010) (Fig. 6B). Notably, the metabolic pathway of phenylalanine involves the formation of phenylacetic acid (Yang et al. 2011) and phenylacetaldehyde, which can be linked to specific enzymes to establish a comprehensive metabolic pathway. This pathway can be divided into two primary directions: the first involves the conversion of phenylalanine into phenylglyoxal in the presence of EC: 4.1.1.28 (aromatic-l-amino-acid decarboxylase), followed by the production of phenylacetaldehyde through the action of EC: 1.4.3.21 (primary-amine oxidase). The second direction involves the formation of phenylalanine into 2-phenylethylamide in the presence of EC: 1.11.1.21 (catalase-peroxidase), followed by the conversion to phenylacetic acid by EC: 3.5.1.4 (amidase). Floral and fruity aromas in CTLs primarily originate from esters and aromatic compounds. Aromatic compounds are characterized by floral, fruity, and sweet scents, with distinct aromas, high boiling points, and limited volatility, making them crucial contributors to the flavor composition of CTLs. Phenylglyoxal, found in the phenylalanine metabolic pathway, is a significant aromatic flavor compound in CTLs, contributing floral and fruity aromas. Consequently, the metabolism of phenylalanine in CTLs can generate floral and fruity aromas in cigars.

Cembranoid diterpenes, commonly found in plants, are synthesized from geranylgeranyl diphosphate (GGPP) through the catalytic action of cembratrienol synthase (CBTS) and cytochrome P450 hydroxylase (CYP450) in the 1-deoxy-d-xylulose-5-phosphate pathway (DXP). GGPP, an intermediate in the DXP pathway of plant terpene metabolism, is synthesized from IPP and DMAPP (Fig. 6C). In tobacco, the biosynthesis of cyproterane diterpenes occurs in two steps: firstly, GGPP provides the cyproterane skeleton structure for the formation of cembratrienol (CBT-ol), which undergoes cyclization to form α- and *β*-CBT-ol in the presence of CBTS (Guo and Wagner 1995); secondly, through the action of CYP450, the sixth carbon of CBT-ol can be degraded to produce various aroma components such as ketones and their derivatives during the growth and processing of tobacco leaves (Wang et al. 2001). Ketones themselves possess a pleasant aroma reminiscent of carrots, licorice, and tea. With further modification and aging of tobacco, ketones can undergo degradation to ketofurans and ketone oxides, which significantly contribute to the flavor of cigarettes. Ketone oxides, for example, impart a subtle nutty and sweet aroma, enhancing the overall aroma profile of cigarettes with notes of cocoa and mild tobacco.

## Discussion

Moisture, reducing sugar, total sugar, and total acid all had different impacts on different VOCs during CTLs fermentation due to the synergistic actions of several microbes. Variations in microbial communities were caused by differences in pile technical operations spanning phases 1, 2, and 3. This phenomenon is caused by non-enzymatic browning interactions between specific sugars and amino acids, which result in the creation of sugar-amino acid condensates. As the temperature increased, enzyme activity in tobacco leaves rose, accelerating and intensifying the browning reactions. Consequently, the sugar-amino acid condensates were more completely converted into aldehydes and ketones, as illustrated in Fig. 1. Furthermore, the degradation of chlorophyll during fermentation emerged as a significant factor influencing the color of CTLs. As the industrial fermentation process advanced, there were slight fluctuations in the color values. The regression analysis presented in Figure S3 further demonstrated that ▲E* accounted for 12.00%, 14.20%, 12.10%, and 16.60% of the variation in moisture, total sugar, reducing sugar, and total acid in tobacco leaves, respectively.

Extensive research has been conducted to unravel the biochemical foundations behind the distinctive and intense flavor of CTLs (Liu et al. 2021, 2022; Zheng et al. 2022a, 2022b). Ketones made up the majority of the detected substances in our investigation, followed by aldehydes and alkanes. This diversity of chemicals closely resembles the aromatic composition of cigars, implying that the cigar filler is a direct source of these aromatic compounds. Notably, we also observed variations in flavor compounds across different fermentation stages, as evidenced by the heatmap and PLS-DA results. Specifically, we found that these compounds exhibited lower diversity and abundance during phase 2 of fermentation. Ketones, being the most pivotal flavoring compounds in cigars, significantly contribute to the mellow, sweet, and roasted characteristics of the cigar’s profile, ultimately enhancing the smoking experience and overall quality (Zheng et al. 2022a).

The predominant bacterial phyla identified in our study, namely Firmicutes, Proteobacteria, and Actinobacteria, have consistently been observed in various studies examining the tobacco leaf microbiome (Fig. 2). These phyla are recognized to play an important role in carbon degradation processes related to starch, xylan, and cellulose absorption. They act as decomposers during tobacco fermentation, breaking down complex macromolecules like cellulose, pectin, and starch into simpler sugars like glucose, fructose, and maltose (Costa et al. 2020). Throughout the fermentation of CTLs, the microbial community changes due to environmental factors such as moisture content and high acidity. As a result, microorganisms that are ill-suited for fermentation conditions, including *Corynebacterium*, *Pseudomonas*, *Staphylococcus*, and *Colletotrichum*, gradually diminish in abundance, leading to a significant loss of biodiversity. It is worth noting that *Corynebacterium*, known for its alkaline resistance, can be a representative genus during fermentation (Hirota et al. 2013; Pang et al. 2020). This characteristic may contribute to its ability to withstand environmental changes during fermentation. Importantly, these changes in microbial composition have implications for the alteration of aroma compounds in CTLs following fermentation (Guo et al. 2019; Alreshidi et al. 2020; Li et al. 2022). *Pseudomonas* has been extensively studied for its role in nicotine degradation and shows potential for regulating the strength of tobacco and improving the quality of upper leaves (Zhong et al. 2010; Zhao et al. 2012). *Staphylococcus* is involved in lipid metabolism and can produce fatty acids that undergo further degradation to form aromatic compounds such as aldehydes and ketones (Costa et al. 2020). *Colletotrichum* can influence the coordination of the main chemical components in CTLs. In contrast, the functional microorganisms that are well-suited for fermentation, such as *Aerococcus* and *Cladosporium*, were found to be enriched during the fermentation process (Fig. 3). *Aerococcus*, known for its salt tolerance and resistance, has been reported as a dominant microorganism in the curing of jatropha shoots. It has also been detected in studies on CTLs, where it utilizes reducing sugars, malic acid, and citric acid present in tobacco leaves to increase temperature and pH, thereby promoting the growth of salt-tolerant and resistant *Corynebacterium* (Li et al. 2020). *Cladosporium* interacts with other bacterial and fungal microflora (Figure S6) and plays a vital role in secreting key enzymes such as amylase and protease, which are responsible for degrading macromolecules and producing characteristic aroma components (Zhang et al. 2018, 2021a). Based on changes in *α*-diversity, the complete fermentation process of CTLs may be separated into three stages (Figure S5). Moisture regaining and drying are two crucial steps in this process. The results of the indicator analysis align well with the diversity of microbial communities, indicating that *Aerococcus*, *Corynebacterium*, *Pseudomonas*, *Staphylococcus*, *Xeromyces*, *Colletotrichum*, and *Nicotiana* all contribute significantly to distinguishing phases 1, 2, and 3. The dominant microorganisms, such as *Aerococcus*, *Corynebacterium*, and *Staphylococcus*, may influence the microbial community structure through enzyme production. It is worth noting that *Xeromyces* has been reported as the most dominant genus in moldy tobacco, suggesting its potential for causing mold (Zhou et al. 2022). Therefore, phase 2, following the rewetting process, maybe a critical stage where tobacco is susceptible to mold growth, necessitating the implementation of anti-mold measures.

The results of Pearson correlation analysis revealed significant correlations between microbial diversity and the presence of ketones and aldehydes, as indicated by the bacterial Shannon index and fungal Shannon index (Figure S7). The most prevalent compounds detected in tobacco leaves are ketones and aldehydes. Aldehydes and ketones have aromatic qualities due to the presence of carbonyl groups in their chemical structure. Many compounds containing carbonyl groups exhibit pleasant aromas. For instance, *β*-damascenone has a powerful rose and fruity aroma, while megastigmatrienone gives tobacco leaves a woody and floral scent. Furthermore, the correlation analysis demonstrated that these VOCs exhibited a stronger correlation with the bacterial Shannon index compared to the fungal Shannon index. This observation further supports the notion that bacterial microorganisms exert a stronger influence on the production of aromatic components in CTLs during fermentation compared to fungal microorganisms, and previous studies align with our findings (Hu et al. 2019). Several dominant genera of CTLs, namely *Pseudomonas*, *Staphylococcus*, and *Stenotrophomonas*, exhibited positive correlations with various aldehydes and ketones. HCA also revealed that aldehydes and ketones were predominantly clustered in clusters 1 and 2. Moreover, VPA highlighted those environmental factors played a pivotal role in shaping the changes in microbial community structure. Different industrial processes introduced alterations in the environment and chemical composition of the CTLs fermentation pile, thereby driving microbial community succession (Fig. 5). Furthermore, we noted that phase 1 was distinguished by the concentration of several important enzymes involved in the synthetic degradation processes of carotenoids, phenylalanines, and cypressanes. The interaction among microorganisms such as *Aerococcus*, *Corynebacterium*, and *Cladosporium* facilitated the degradation of macromolecules and the production of characteristic aroma components (Zhang et al. 2018, 2021a). This may also explain the higher variety and quantity of VOCs in phase 1 compared to the other phases. However, for the quality of CTLs, achieving a harmonious blend of chemical and aromatic components relies on the subsequent deep fermentation stage, which is crucial for attaining optimal draw quality and overall balance.

In conclusion, this is the first thorough study of microbial populations, physicochemical parameters, and flavor compounds during the industrial fermentation of CTLs, focusing on the various phases of phase 1, phase 2, and phase 3. Notably, moisture regaining and drying operations play critical roles in the industrial production process, signifying the transition from phase1 to phase 2 and phase 2 to phase 3, respectively. Significant variations among microorganisms were primarily observed in phase1 and phase 2. In phase 2, the elevated levels of total acid led to the rapid dominance of *Aerococcus* as the prevailing bacterial species. Conversely, the fungal microbial community structure remained relatively stable, with changes being less pronounced compared to bacteria. Throughout each stage, *Aspergillus*, *Alternaria*, and *Cladosporium* consistently constituted the major fungal groups. Correlation analysis and indicator analysis revealed the prominent role of specific bacterial genera, such as *Pseudomonas*, *Staphylococcus*, and *Stenotrophomonas*, as well as fungal genera including *Colletotrichum* and *Cumuliphoma*, in driving the bioconversion of flavor compounds, particularly ketones, during CTLs fermentation. These differential microorganisms represent key players in the complex process of VOC formation. The findings of this study shed light on the core microbial communities involved and their contributions to VOCs production during the industrial fermentation of CTLs. The results also provide the theoretical foundation for enhancing cigar leaves quality through targeted microbial enhancement techniques.

## Supplementary Information

Below is the link to the electronic supplementary material.

Supplementary file1 (PDF 11270 KB)

## Data Availability

The sequence data of this study have been deposited in the CNCB BioProject database under BioProject accession numbers PRJCA017815 and PRJCA017819.
