# Supplementary material for: Analyzing microbial community and volatile compound profiles in the fermentation of cigar tobacco leaves
Source: Appl Microbiol Biotechnol. 2024 Feb 29;108:243. doi: 10.1007/s00253-024-13043-3 (PMC10904427; doi:10.1007/s00253-024-13043-3)
Supplement: Supplementary file 1 [file 253_2024_13043_MOESM1_ESM.pdf]

**Analyzing Microbial Community and Volatile Compound Profiles in the Fermentation of Cigar Tobacco Leaves**

Mingzhu Zhang[#1] · Dongfeng Guo[#2*] · Haiqing Wang[1] · Guanglong Wu[1] · Yaqi Shi[2] · Jinlong Zhou[2] · Eryong Zhao[1] · Tianfei Zheng[1,2] · Xingjiang Li[1*]

1 Key Laboratory for Agricultural Products Processing, School of Food and Biological Engineering, Hefei University of Technology, Hefei, Anhui, China

2 China Tobacco Anhui Industry Co., Ltd., Hefei, Anhui, China

# Mingzhu Zhang and Dongfeng Guo contributed equally to this work.

**Correspondence**

Xingjiang Li, Key Laboratory for Agricultural Products Processing, School of Food and Biological Engineering, Hefei University of Technology, Danxia Road 485#, Zip Code 230601, Hefei City, Anhui Province, China. E-mail: lixingjiang@hfut.edu.cn

Dongfeng Guo, Anhui China Tobacco Industry Co., Ltd., Huangshan Road 606#, Zip Code 230088, Hefei City, Anhui Province, China. E-mail: gdf0221@163.com

**Table S1 Information of CTLs**

| Sample | Fermentation stage |
|---|---|
| UFCL | CTLs before moisture regaining |
| MRCL | CTLs after moisture regaining |
| FCL | Fermented CTLs after moisture regaining |
| FTCL | CTLs after the first turn |
| STCL | CTLs after the second turn |
| DCL | CTLs after fermentation and drying |

**Table S2 Index of microbial diversity in CTLs at different fermentation stages**

| Group | Phase1 | Phase2 | Phase3 |
|---|---|---|---|
| **Bacteria** | | | |
| Raw Reads | 193755.00 ± 24182.00 | 166112.00 ± 34535.00 | 157430.00 ± 56598.00 |
| Clean Reads | 193482.00 ± 24143.00 | 165834.00 ± 34471.00 | 157226.00 ± 56519.00 |
| Sobs | 75.33 ± 6.66 | 63.67 ± 14.03 | 67.67 ± 16.17 |
| Shannon | 2.59 ± 0.28 | 1.28 ± 0.31 | 0.68 ± 0.02 |
| Simpson | 0.75 ± 0.05 | 0.38 ± 0.10 | 0.16 ± 0.01 |
| Chao | 91.87 ± 12.23 | 74.31 ± 14.84 | 73.13 ± 14.14 |
| Ace | 89.75 ± 8.94 | 75.57 ± 13.33 | 74.87 ± 11.33 |
| Goods coverage | 1.00 ± 0.00 | 1.00 ± 0.00 | 1.00 ± 0.00 |
| **Fungi** | | | |
| Raw Reads | 129846.00 ± 5687.00 | 128112.00 ± 5919.00 | 126690.00 ± 1284.00 |
| Clean Reads | 129647.00 ± 5675.00 | 127623.00 ± 5856.00 | 126540.00 ± 1285.00 |
| Sobs | 75.00 ± 1.00 | 48.25 ± 4.39 | 52.00 ± 4.58 |
| Shannon | 2.66 ± 0.30 | 2.74 ± 0.31 | 2.11 ± 0.48 |
| Simpson | 0.75 ± 0.08 | 0.75 ± 0.08 | 0.64 ± 0.14 |
| Chao | 82.25 ± 6.53 | 54.45 ± 6.69 | 56.86 ± 6.18 |

| | | | |
|---|---|---|---|
| Ace | 82.51 ± 8.12 | 54.49 ± 6.42 | 57.70 ± 4.86 |
| Goods coverage | 1.00 ± 0.00 | 1.00 ± 0.00 | 1.00 ± 0.00 |

**Table S3 The 34 major volatile compounds (VIP>1.0)**

| NO. | CAS | Description | Compounds | Threshold value (μg/mL) |
|---|---|---|---|---|
| 1 | 432-25-7 | Tropical saffron herbal clean rose oxide sweet tobacco damascone fruity | Beta-cyclocitral | 0.003 |
| 2 | 112-31-2 | Floral, fried, orange peel, penetrating, tallow | Decyl aldehyde | 0.0001 |
| 3 | 116-26-7 | Herb | Safranal | / |
| 4 | 122-78-1 | Berry, geranium, honey, nut, pungent | Phenylacetaldehyde | 0.001 |
| 5 | 40702-26-9 | Apple, sweet, fruity | 1,3,4-Trimethyl-3-cyclohexen-1-carboxaldehyde | / |
| 6 | 98-86-2 | Almonds, flower, meat, must | Acetophenone | 0.065 |
| 7 | 78-59-1 | Cedarwood, spice | Isophorone | 11 |
| 8 | 1125-21-9 | Floral | 2,6,6-Trimethyl-2-cyclohexene-1,4-dione | 0.025 |
| 9 | 10519-33-2 | Savory | 3-Decen-2-one | / |
| 10 | 491-09-8 | Mint, sharp | 3-Methyl-6-(1-methylethylidene)-2-cyclohexen-1-on | / |
| 11 | 39917-38-9 | Flowery, woody | 1-(3-Hydroxy-adamantan-1-yl)-ethanone | / |
| 12 | 83406-41-1 | Tea | 1,8(2H,5H)-Naphthalenedione | / |
| 13 | 5835-18-7 | Sweet, herb, tobacco | 2,5,5,8a-Tetramethyl-2,3,4,4a,6,8-hexahydrochromen-7-one | / |
| 14 | 2047-21-4 | Sweet, flowery, and woody | 1-(3,4,5-Trimethylphenyl) ethanone | 0.021 |
| 15 | 1117-52-8 | flower ether | Farnesylacetone | 0.1 |
| 16 | 54410-98-9 | Sweet, fruity, peach and almond. | 1-Nonene,4,6,8-trimethyl- | / |
| 17 | 55702-54-0 | / | 3-Cyclohexylpent-4-en-2-one | / |
| 18 | 16778-27-1 | Fruit | Tetrahydro-actinidiolide | 2.8 |
| 19 | 1120-21-4 | Fruity | Undecane | 10 |
| 20 | 17301-33-6 | Fruity | 4,8-Dimethylundecane | / |
| 21 | 112-40-3 | alkane | Dodecane | 13000 |
| 22 | 96-08-2 | Mentholic | 1-Methyloxiranyl)-7-oxabicyclo [4.1.0] heptane | / |
| 23 | 3168-90-9 | Nutty, sweet | 1-(2-Methyl-1-cyclopentenyl) ethanone | / |
| 24 | 515-13-9 | Sweet | Beta-elemene | / |
| 25 | 62108-28-5 | Fragrance | 4,8-Dimethyl-1,7-nonadiene | / |

| | | | | |
|---|---|---|---|---|
| 26 | 10482-56-1 | Lilac floral terpenic | (-)-Alpha-terpineol | 1.2 |
| 27 | 128-37-0 | Toasted cereal | 2,6-Di-tert-butyl-4-methylphenol | 1 |
| 28 | 551-45-1 | / | 3-Isopropylphenol | / |
| 29 | 350-03-8 | Savory | 3-Acetylpyridine | 0.5 |
| 30 | 15469-77-9 | / | 3-Decenoic acid | / |
| 31 | 532-12-7 | Nutty, sweet | Myosmine | / |
| 32 | 77-53-2 | Cedarwood woody dry sweet soft | Cedrol | / |
| 33 | 91-22-5 | Medical musty tobacco rubber earthy | Quinoline | 0.71 |
| 34 | 120-72-9 | Burnt, mothball | Indole | 0.023 |

Odour descriptions are based on The Good Scents Company (http://www.thegoodscentscompany.com/search2.html) and Flavor Ingredient Library (https://www.femaflavor.org/flavor-library). Threshold value reference L.J. van Gemert, Odour thresholds, Compilations of odour threshold values in air, water and other media, Oliemans Punter & Co. (2011).

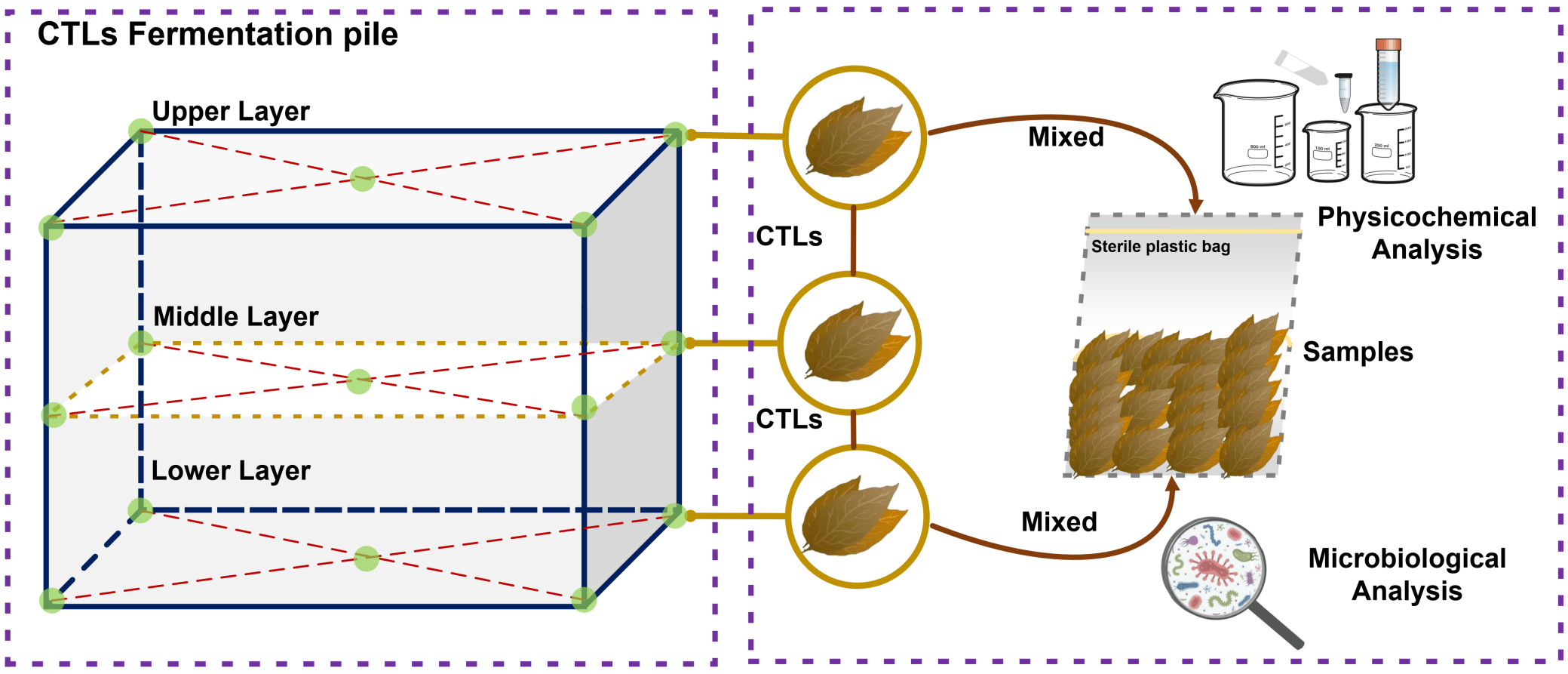


**Figure S1** Cigar tobacco leaves (CTLs) sampling diagram

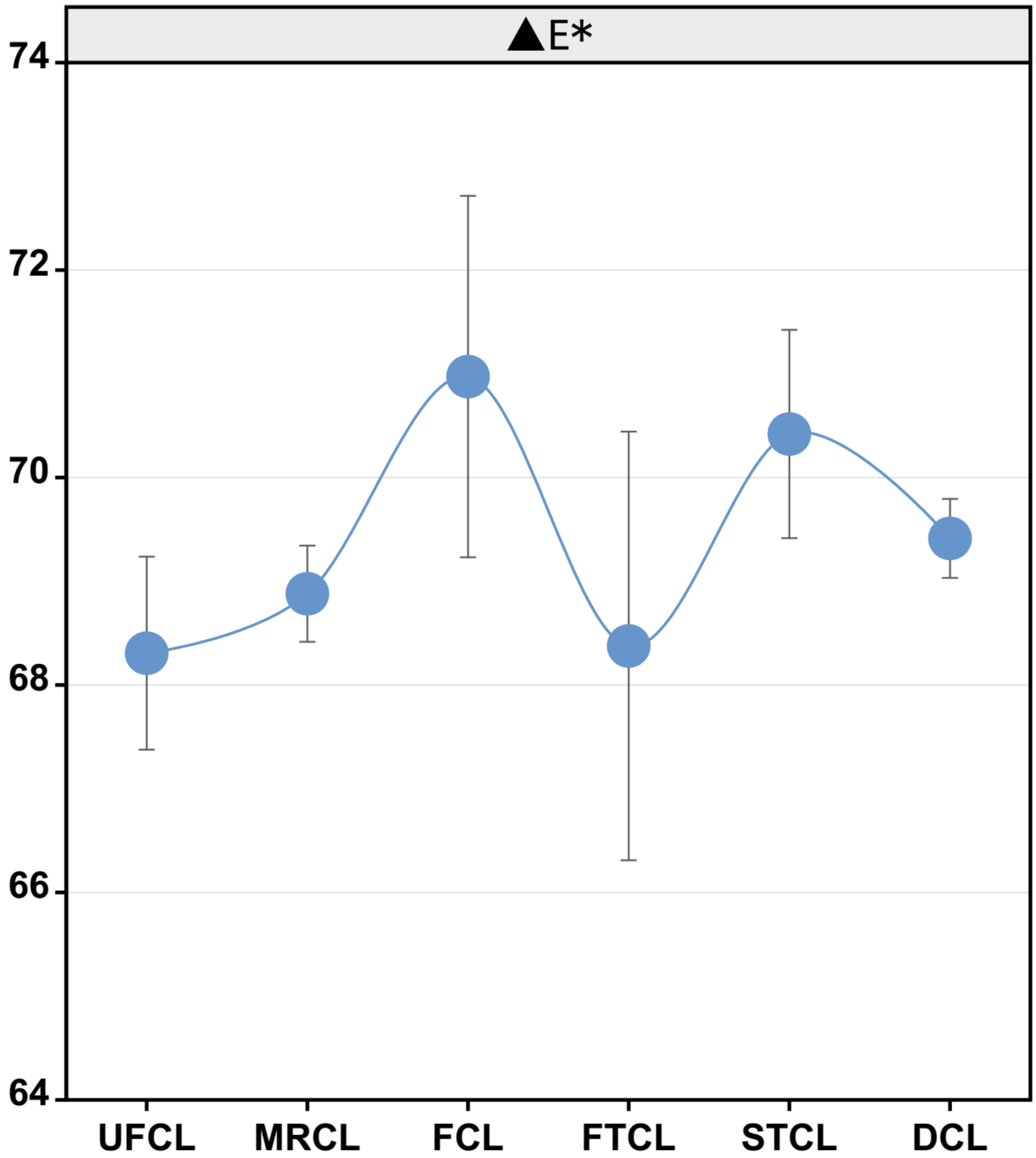


**Figure S2** Changes in the ▲E* of CTLs in different fermentation stages

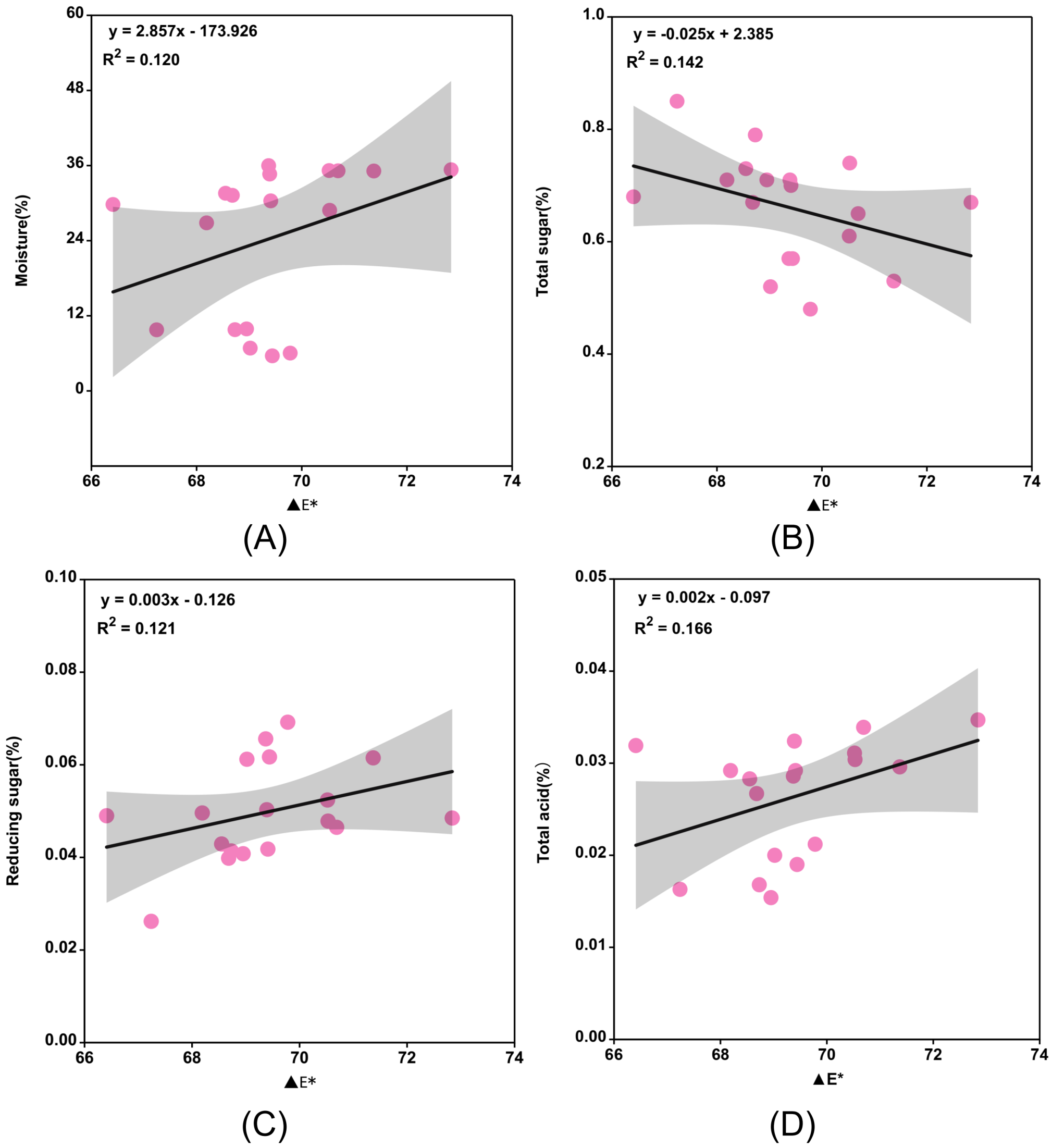


**Figure S3** Regression analysis of physicochemical parameters and ▲E* in CTLs during industrial fermentation

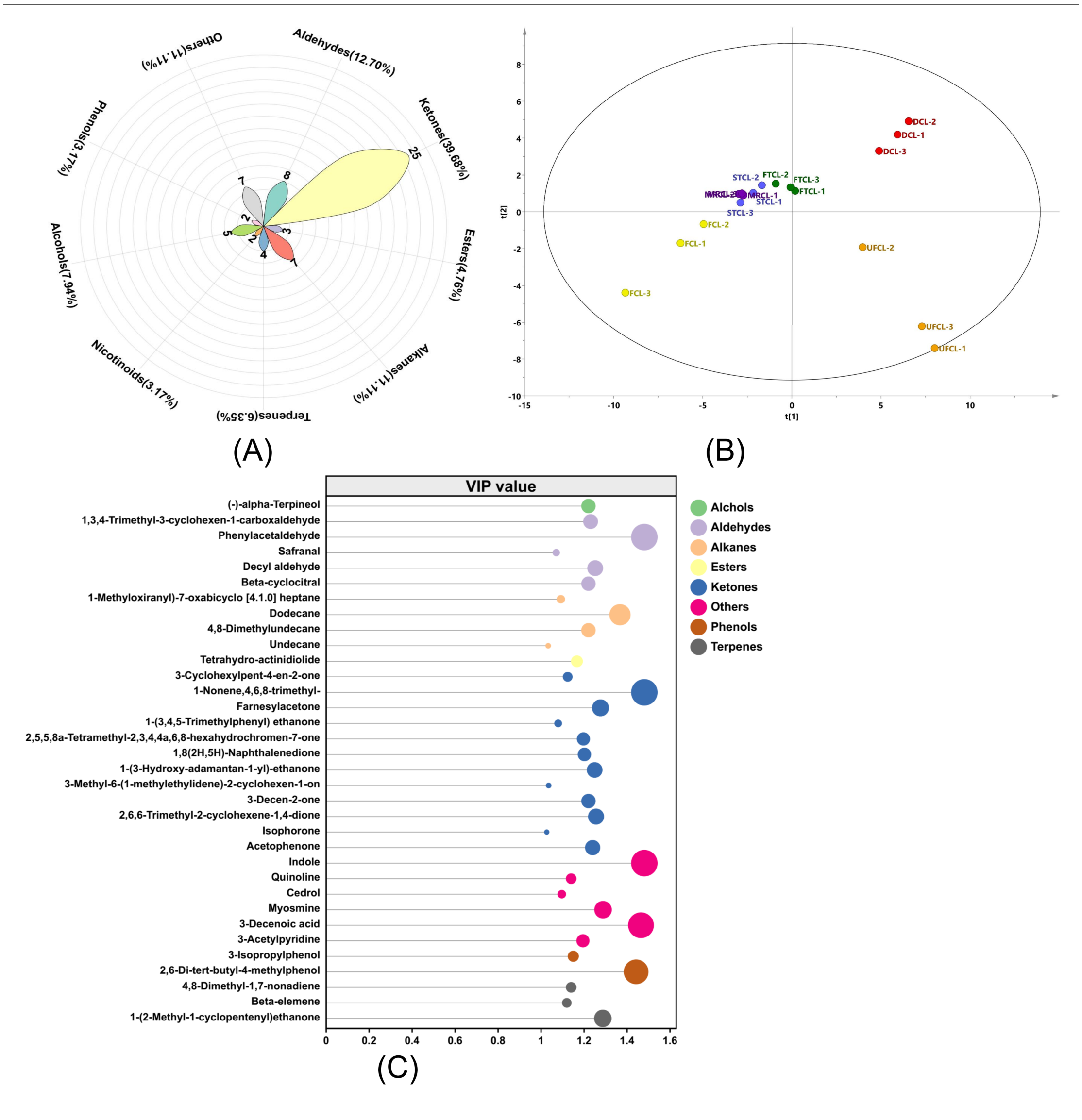


**Figure S4** The categories of flavor substances (A), PCA diagram of flavor substances (B) and flavor substances with VIP value >1 (C)

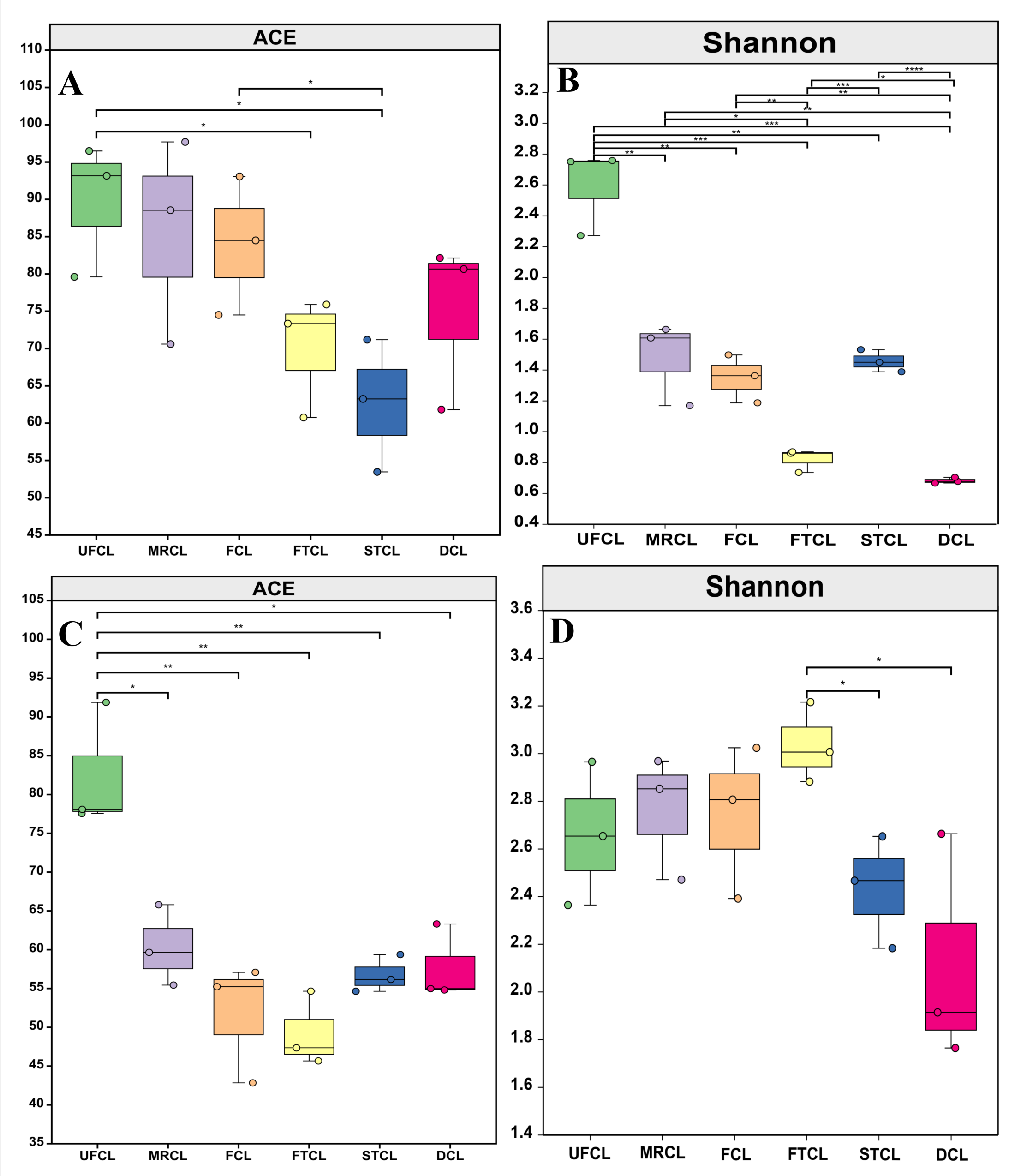


**Figure S5** Alpha diversity analysis of bacterial and fungal microorganisms of CTLs

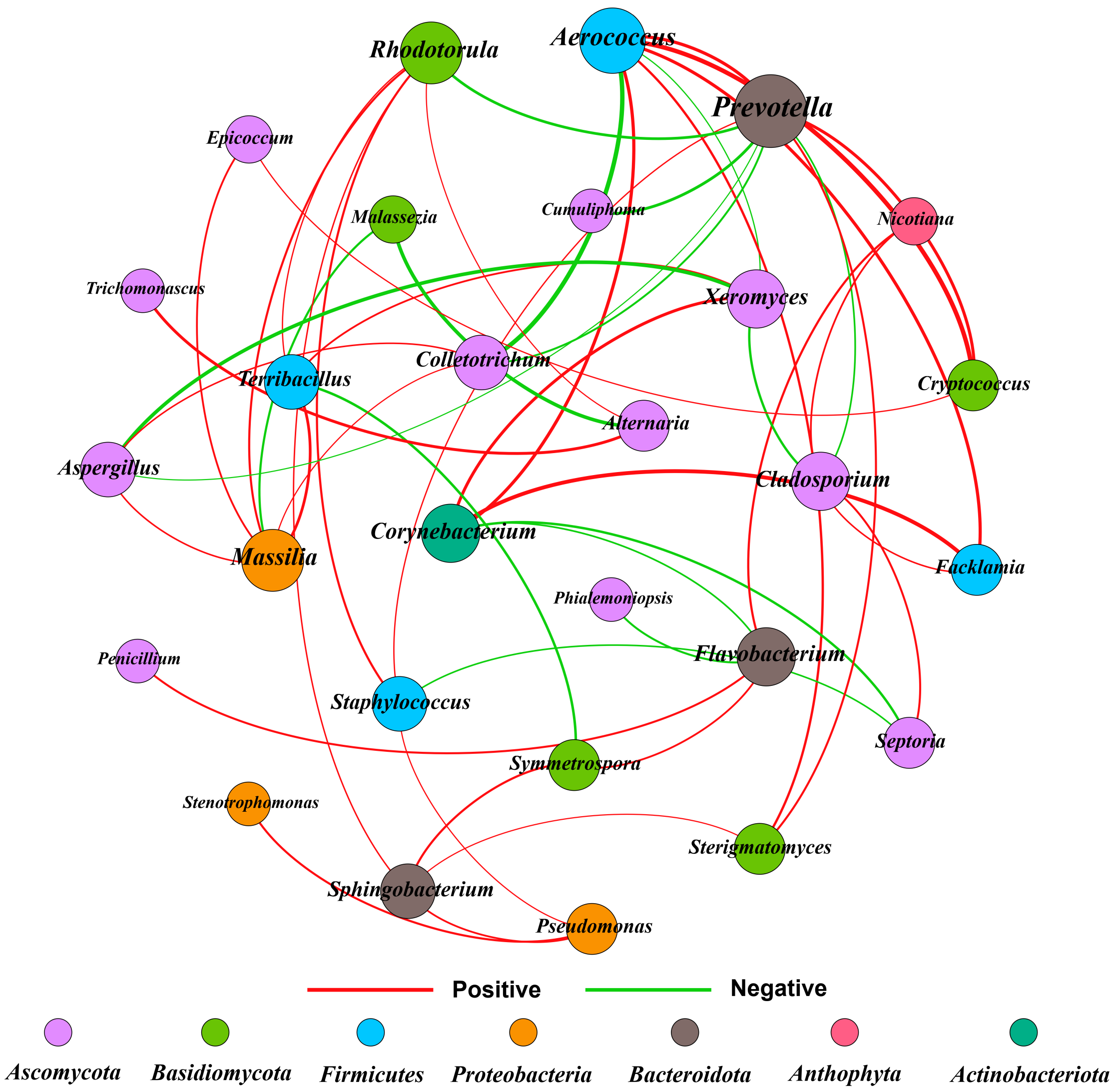


**Figure S6** The pearson's correlation network between selected bacteria and fungi, different colors represent different phylum of bacteria and fungi, the red line represents the positive correlation and the green represents the negative correlation ($p<0.05$)

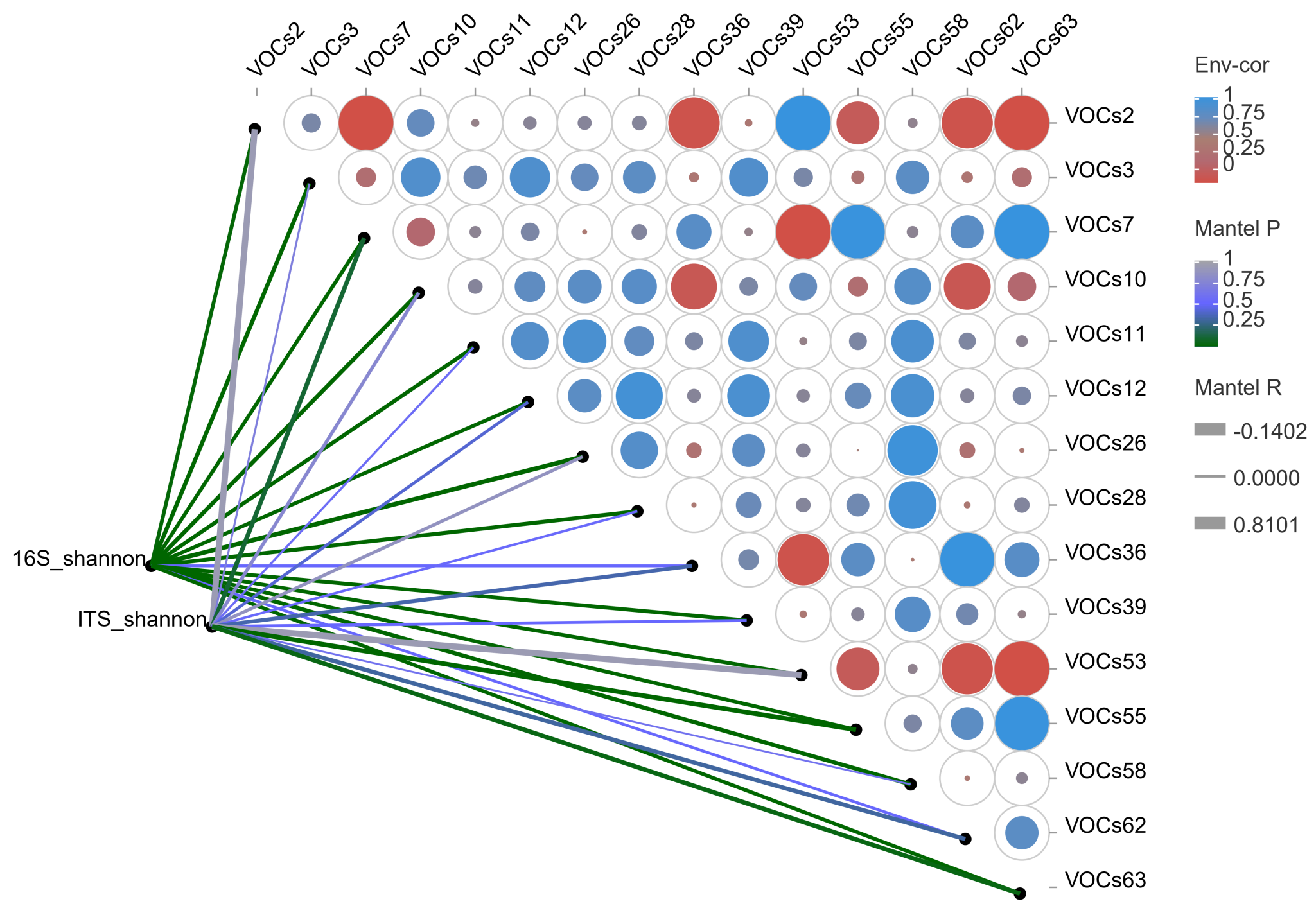


**Figure S7** The relationship between bacteria, fungi and VOCs was analyzed by heat map and network map, VOCs2, beta-cyclocitral; VOCs3, decyl aldehyde; VOCs7, phenylacetaldehyde; VOCs10, acetophenone; VOCs11, isophorone; VOCs12, 2,6,6-trimethyl-2-cyclohexene-1,4-dione; VOCs26, 1-(3,4,5-trimethylphenyl) ethenone; VOCs28, farnesylacetone; VOCs36, tetrahydro-actinidiolide; VOCs39, undecane; VOCs53, (-)-alpha-terpineol; VOCs55, 2,6-di-tert-butyl-4-methylphenol; VOCs58, 3-acetylpyridine; VOCs62, quinoline and VOCs63, indole
