# Supplementary material for: Analyzing microbial community and volatile compound profiles in the fermentation of cigar tobacco leaves
Source: Appl Microbiol Biotechnol. 2024 Feb 29;108(1):243. doi: 10.1007/s00253-024-13043-3 (PMC10904427; doi:10.1007/s00253-024-13043-3)
Supplement: Supplementary file 1 — Supplementary file1 (PDF 11270 KB) [file 253_2024_13043_MOESM1_ESM.pdf]

Analyzing Microbial Community and Volatile Compound Profiles in the Fermentation of Cigar Tobacco Leaves

Mingzhu Zhang[#1] · Dongfeng Guo[#2*] · Haiqing Wang[1] · Guanglong Wu[1] · Yaqi Shi[2] · Jinlong Zhou[2] · Eryong Zhao[1] · Tianfei Zheng[1,2] · Xingjiang Li[1*]

1 Key Laboratory for Agricultural Products Processing, School of Food and Biological Engineering, Hefei University of Technology, Hefei, Anhui, China

2 China Tobacco Anhui Industry Co., Ltd., Hefei, Anhui, China

# Mingzhu Zhang and Dongfeng Guo contributed equally to this work.

Correspondence

Xingjiang Li, Key Laboratory for Agricultural Products Processing, School of Food and Biological Engineering, Hefei University of Technology, Danxia Road 485#, Zip Code 230601, Hefei City, Anhui Province, China. E-mail: lixingjiang@hfut.edu.cn

Dongfeng Guo, Anhui China Tobacco Industry Co., Ltd., Huangshan Road 606#, Zip Code 230088, Hefei City, Anhui Province, China. E-mail: gdf0221@163.com

Table S1 Information of CTLs

Sample	Fermentation stage
UFCL	CTLs before moisture regaining
MRCL	CTLs after moisture regaining
FCL	Fermented CTLs after moisture regaining
FTCL	CTLs after the first turn
STCL	CTLs after the second turn
DCL	CTLs after fermentation and drying

Table S2 Index of microbial diversity in CTLs at different fermentation stages

Group	Phase1	Phase2	Phase3
**Bacteria**			
Raw Reads	193755.00 ± 24182.00	166112.00 ± 34535.00	157430.00 ± 56598.00
Clean Reads	193482.00 ± 24143.00	165834.00 ± 34471.00	157226.00 ± 56519.00
Sobs	75.33 ± 6.66	63.67 ± 14.03	67.67 ± 16.17
Shannon	2.59 ± 0.28	1.28 ± 0.31	0.68 ± 0.02
Simpson	0.75 ± 0.05	0.38 ± 0.10	0.16 ± 0.01
Chao	91.87 ± 12.23	74.31 ± 14.84	73.13 ± 14.14
Ace	89.75 ± 8.94	75.57 ± 13.33	74.87 ± 11.33
Goods coverage	1.00 ± 0.00	1.00 ± 0.00	1.00 ± 0.00
**Fungi**			
Raw Reads	129846.00 ± 5687.00	128112.00 ± 5919.00	126690.00 ± 1284.00
Clean Reads	129647.00 ± 5675.00	127623.00 ± 5856.00	126540.00 ± 1285.00
Sobs	75.00 ± 1.00	48.25 ± 4.39	52.00 ± 4.58
Shannon	2.66 ± 0.30	2.74 ± 0.31	2.11 ± 0.48
Simpson	0.75 ± 0.08	0.75 ± 0.08	0.64 ± 0.14
Chao	82.25 ± 6.53	54.45 ± 6.69	56.86 ± 6.18

Ace	82.51 ± 8.12	54.49 ± 6.42	57.70 ± 4.86
Goods coverage	1.00 ± 0.00	1.00 ± 0.00	1.00 ± 0.00

Table S3 The 34 major volatile compounds (VIP>1.0)

NO.	CAS	Description	Compounds	Threshold value (μg/mL)
1	432-25-7	Tropical saffron herbal clean rose oxide sweet tobacco damascone fruity	Beta-cyclocitral	0.003
2	112-31-2	Floral, fried, orange peel, penetrating, tallow	Decyl aldehyde	0.0001
3	116-26-7	Herb	Safranal	/
4	122-78-1	Berry, geranium, honey, nut, pungent	Phenylacetaldehyde	0.001
5	40702-26-9	Apple, sweet, fruity	1,3,4-Trimethyl-3-cyclohexen-1-carboxaldehyde	/
6	98-86-2	Almonds, flower, meat, must	Acetophenone	0.065
7	78-59-1	Cedarwood, spice	Isophorone	11
8	1125-21-9	Floral	2,6,6-Trimethyl-2-cyclohexene-1,4-dione	0.025
9	10519-33-2	Savory	3-Decen-2-one	/
10	491-09-8	Mint, sharp	3-Methyl-6-(1-methylethylidene)-2-cyclohexen-1-on	/
11	39917-38-9	Flowery, woody	1-(3-Hydroxy-adamantan-1-yl)-ethanone	/
12	83406-41-1	Tea	1,8(2H,5H)-Naphthalenedione	/
13	5835-18-7	Sweet, herb, tobacco	2,5,5,8a-Tetramethyl-2,3,4,4a,6,8-hexahydrochromen-7-one	/
14	2047-21-4	Sweet, flowery, and woody	1-(3,4,5-Trimethylphenyl) ethanone	0.021
15	1117-52-8	flower ether	Farnesylacetone	0.1
16	54410-98-9	Sweet, fruity, peach and almond.	1-Nonene,4,6,8-trimethyl-	/
17	55702-54-0	/	3-Cyclohexylpent-4-en-2-one	/
18	16778-27-1	Fruit	Tetrahydro-actinidiolide	2.8
19	1120-21-4	Fruity	Undecane	10
20	17301-33-6	Fruity	4,8-Dimethylundecane	/
21	112-40-3	alkane	Dodecane	13000
22	96-08-2	Mentholic	1-Methyloxiranyl)-7-oxabicyclo [4.1.0] heptane	/
23	3168-90-9	Nutty, sweet	1-(2-Methyl-1-cyclopentenyl) ethanone	/
24	515-13-9	Sweet	Beta-elemene	/
25	62108-28-5	Fragrance	4,8-Dimethyl-1,7-nonadiene	/

26	10482-56-1	Lilac floral terpenic	(-)-Alpha-terpineol	1.2
27	128-37-0	Toasted cereal	2,6-Di-tert-butyl-4-methylphenol	1
28	551-45-1	/	3-Isopropylphenol	/
29	350-03-8	Savory	3-Acetylpyridine	0.5
30	15469-77-9	/	3-Decenoic acid	/
31	532-12-7	Nutty, sweet	Myosmine	/
32	77-53-2	Cedarwood woody dry sweet soft	Cedrol	/
33	91-22-5	Medical musty tobacco rubber earthy	Quinoline	0.71
34	120-72-9	Burnt, mothball	Indole	0.023

Odour descriptions are based on The Good Scents Company (http://www.thegoodscentscompany.com/search2.html) and Flavor Ingredient Library (https://www.femaflavor.org/flavor-library). Threshold value reference L.J. van Gemert, Odour thresholds, Compilations of odour threshold values in air, water and other media, Oliemans Punter & Co. (2011).

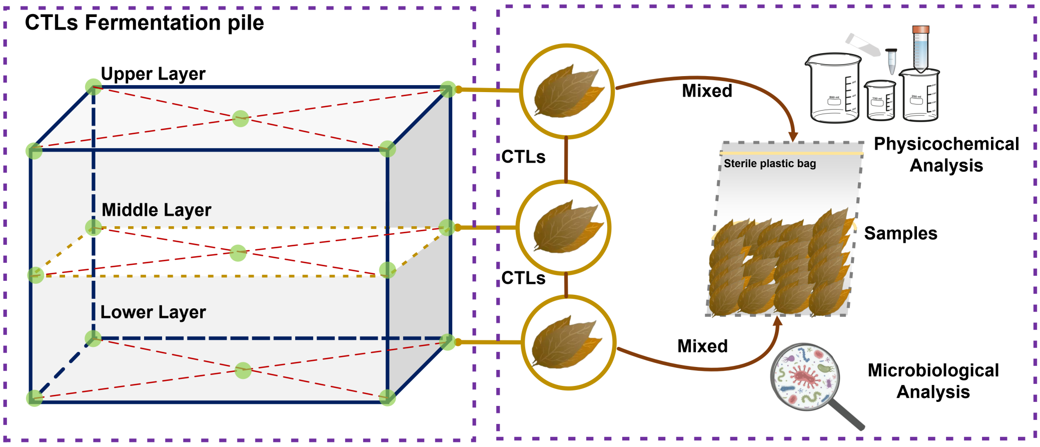

Figure S1 Cigar tobacco leaves (CTLs) sampling diagram

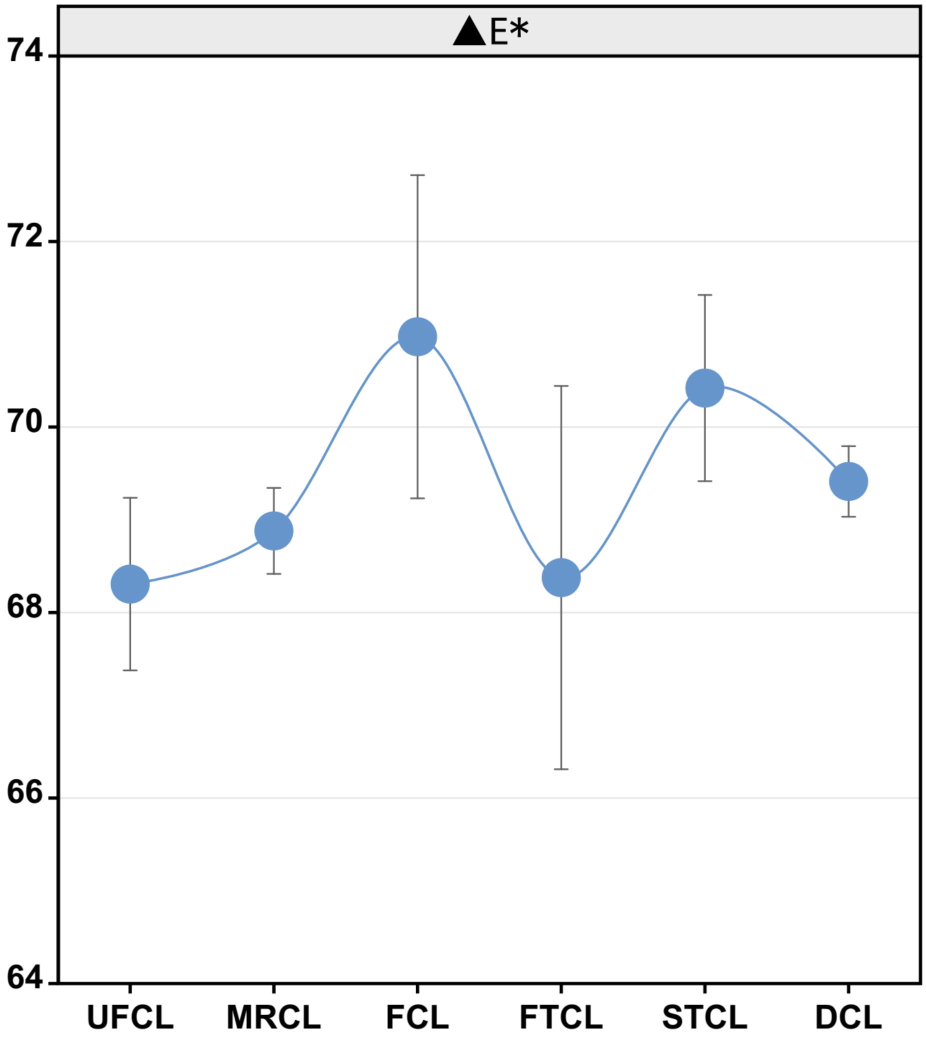

Figure S2 Changes in the ▲E* of CTLs in different fermentation stages

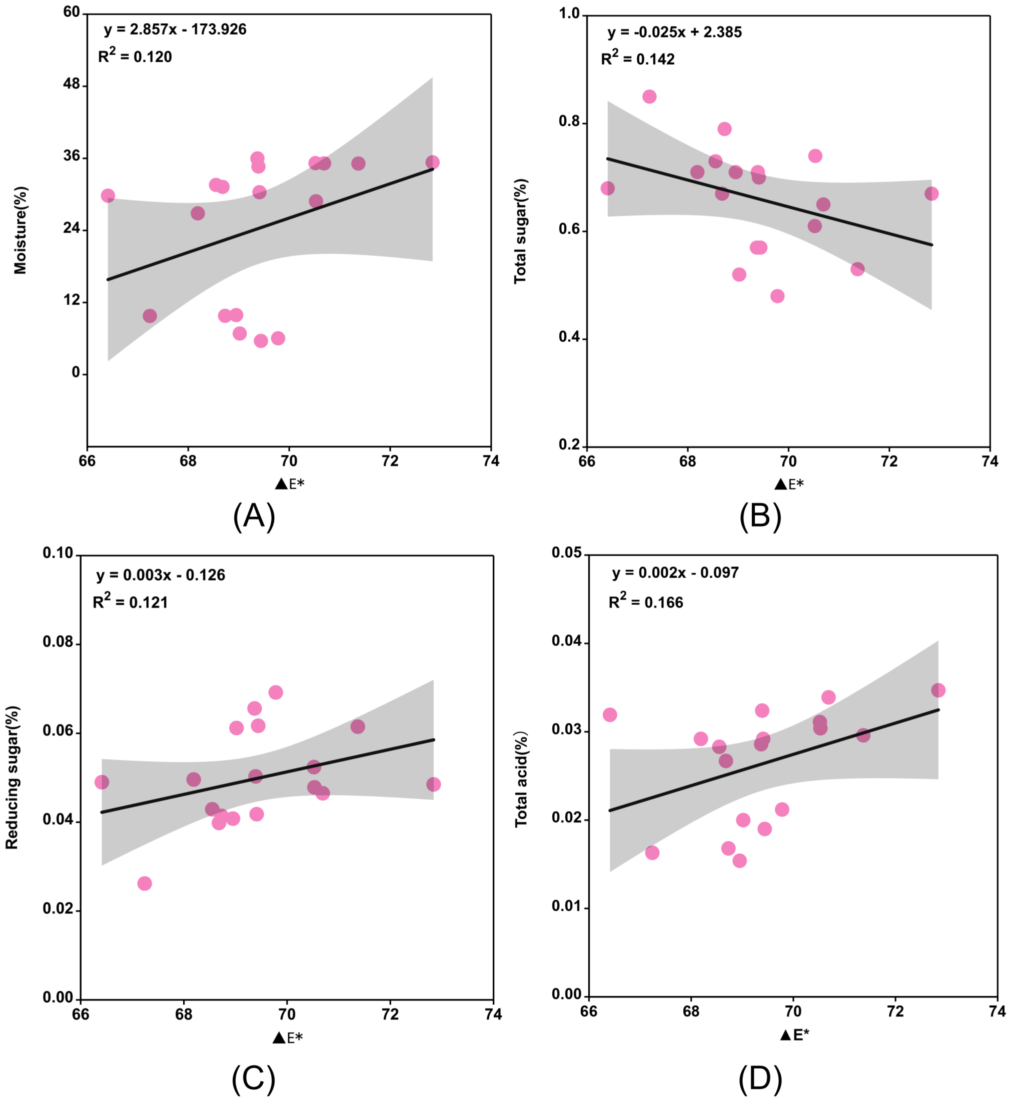

Figure S3 Regression analysis of physicochemical parameters and ▲E* in CTLs during industrial fermentation

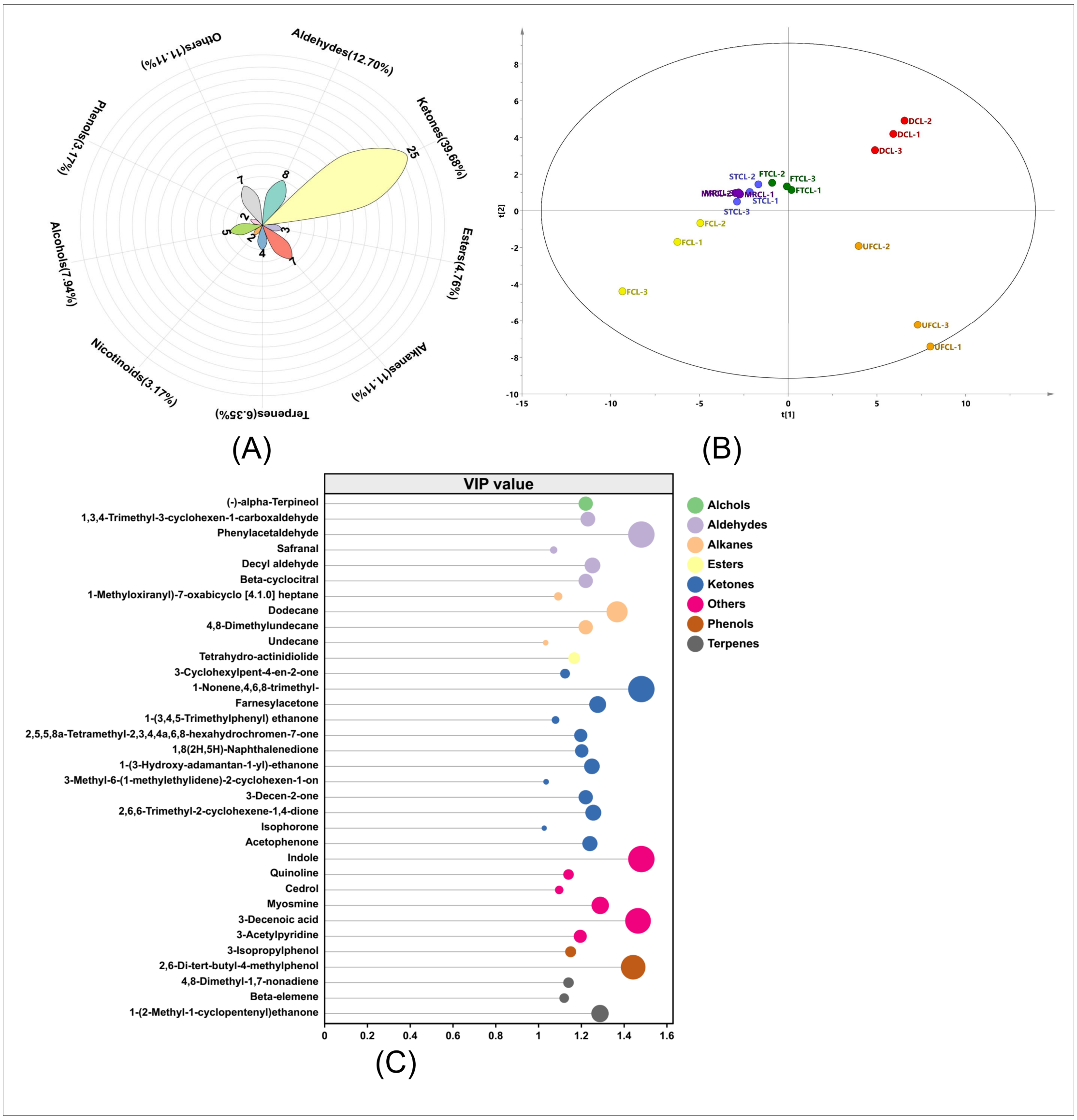

Figure S4 The categories of flavor substances (A), PCA diagram of flavor substances (B) and flavor substances with VIP value >1 (C)

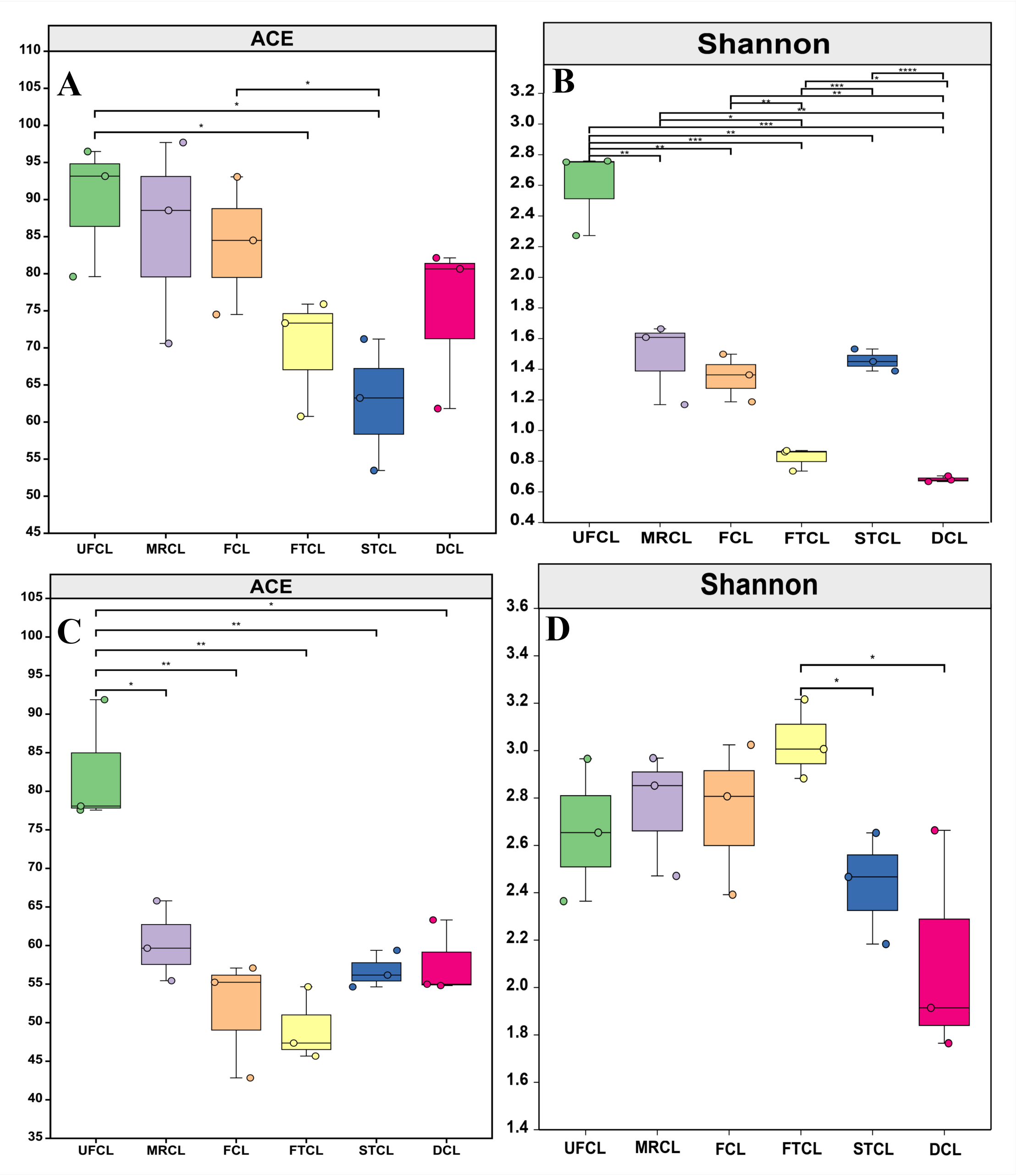

Figure S5 Alpha diversity analysis of bacterial and fungal microorganisms of CTLs

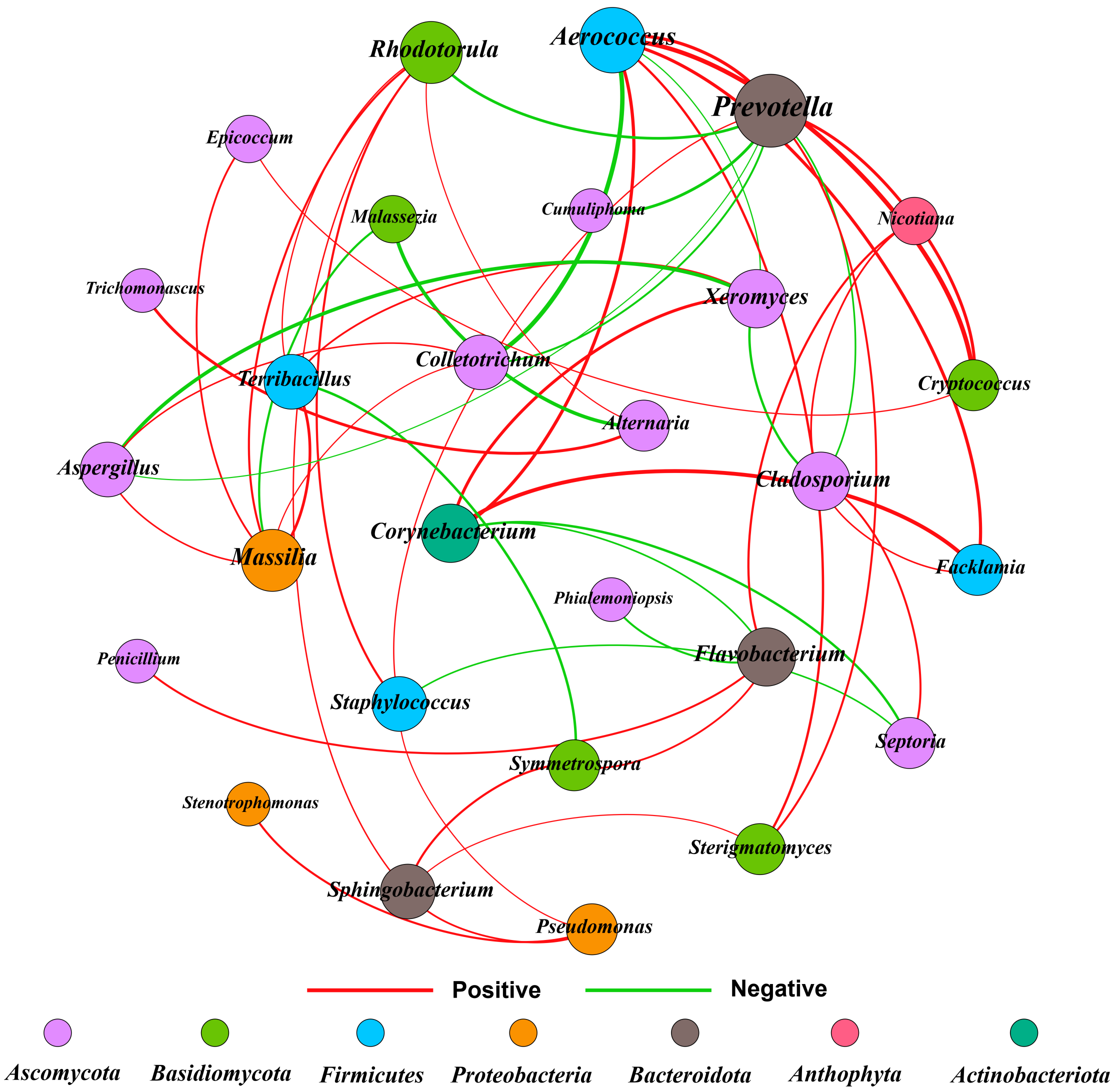

Figure S6 The pearson's correlation network between selected bacteria and fungi, different colors represent different phylum of bacteria and fungi, the red line represents the positive correlation and the green represents the negative correlation ($p<0.05$)

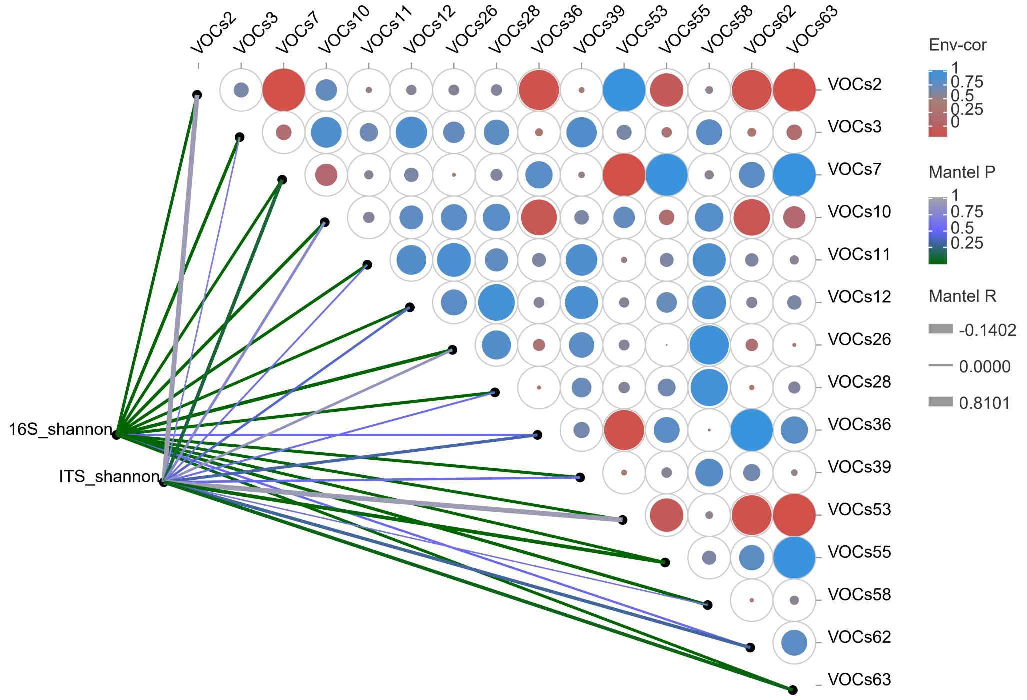

Figure S7 The relationship between bacteria, fungi and VOCs was analyzed by heat map and network map, VOCs2, beta-cyclocitral; VOCs3, decyl aldehyde; VOCs7, phenylacetaldehyde; VOCs10, acetophenone; VOCs11, isophorone; VOCs12, 2,6,6-trimethyl-2-cyclohexene-1,4-dione; VOCs26, 1-(3,4,5-trimethylphenyl) ethenone; VOCs28, farnesylacetone; VOCs36, tetrahydro-actinidiolide; VOCs39, undecane; VOCs53, (-)-alpha-terpineol; VOCs55, 2,6-di-tert-butyl-4-methylphenol; VOCs58, 3-acetylpyridine; VOCs62, quinoline and VOCs63, indole
